# The UN SDGs as a global ‘directive shift’ and the institutionalization of sustainability research

**DOI:** 10.1371/journal.pone.0348507

**Published:** 2026-06-03

**Authors:** Alesia A. Zuccala, Anna Leoncini, Andrea Bonaccorsi

**Affiliations:** 1 Department of Communication, University of Copenhagen, Copenhagen, Denmark; 2 Department of Energy, Systems, Territory and Construction Engineering, University of Pisa, Pisa, Italy; Universidade Aberta Departamento de Ciencias Sociais e de Gestao, PORTUGAL

## Abstract

This paper examines how the UN Sustainable Development Goals (SDGs) shape the institutionalization of sustainability research within scholarly publishing. We argue that the SDGs operate as a globally endorsed form of external research agenda-setting, constituting a “directive shift” in science. Focusing on SDG 04 (Quality Education), SDG 08 (Decent Work and Economic Growth), and SDG 13 (Climate Action), we analyse changes in Scopus-indexed journals from 1990 to 2024. Using large-scale bibliometric data, we classify (*n* = 30,604) journals by activity level, age (newborn, young, mature, established), disciplinarity, publishing model, and long-term survival across publication thresholds (*k* = 1, 3, 5, 10). Results reveal a sustained increase in journal participation related to SDG-related publishing, with pronounced entry surges around major international agreements in 2005 and 2015. Participation is driven primarily by young and mature journals, while established journals contribute a comparatively small share of new entrants. Further analysis of established titles reveals that top-ranked (Q1) core journals are more prominent in SDG 13 than in SDG 04 and SDG 08, suggesting uneven integration across disciplinary hierarchies. Multidisciplinary and open-access journals dominate entry patterns, and survival rates increase at higher publication thresholds, indicating sustained engagement over time. Overall, these structural dynamics suggest that the SDGs operate as a directive shift, contributing to the progressive consolidation of sustainability research within the journal system.

## 1. Introduction

The relationship between scientific research and societal goals has long been debated in science policy and the history of science [1]. A central tension concerns the balance between scientific autonomy and societal direction. While science is often understood as driven by internally defined epistemic challenges, societal needs generate expectations that research agendas should contribute to addressing public problems.

Classic accounts emphasize the internal dynamics of scientific change. Kuhn [2] conceptualized scientific progress as paradigm shifts arising from epistemic anomalies, while Crane [3] and Mullins [4] showed how informal networks of researchers consolidate into recognized specialties and institutionalized fields. Field formation may occur through disciplinary fusion, specialization, or convergence between applied and basic sciences [5], often facilitated by scientific brokers connecting otherwise unconnected actors [6]. In these perspectives, scientific development is largely understood as emerging from within research communities.

At the same time, an extensive body of scholarship highlights the social and institutional embeddedness of science [7,8]. As Funtowicz and Ravetz [9] suggest, the era of insulated “puzzle-solving” has increasingly given way to forms of research shaped by broader societal concerns. External pressures can therefore reshape professional roles [10], challenge disciplinary boundaries [11], or prompt fields to renegotiate legitimacy and resources [12,13]. In some cases, they have catalysed convergence across disciplines, giving rise to new interdisciplinary domains such as the synthetic life sciences [14,15].

Perspectives on scientific change have also informed discussions in science policy about how public authorities might influence the direction of research while preserving scientific autonomy [16–18]. Together, these discussions highlight an evolving balance between internally generated epistemic dynamics and externally articulated societal priorities. This tension was famously articulated by Nelson [19] in *The Moon and the Ghetto*, which posed the question of why science has been able to mobilize extraordinary resources to achieve technical feats such as landing astronauts on the Moon while proving far less effective at addressing persistent societal challenges such as poverty and inequality. The challenge identified by [20].

A prominent contemporary effort to align scientific research with societal priorities can be seen in the United Nations’ Sustainable Development Goals (SDGs). Although sustainability has been present in academic discourse since the 1980s [21], the adoption of the 17 SDGs in 2015 formalized a globally endorsed framework addressing climate change, inequality, poverty, biodiversity loss, and related systemic challenges. Scientists have been explicitly called upon to align research efforts with these objectives. Recent studies have begun to examine how national research systems and scientific communities respond to these goals [22,23]. This can be conceptualised as a “directive shift”: a globally legitimised orientation that guides and encourages the reconfiguration of research priorities across established disciplinary domains.

The present study investigates whether and how such a directive shift is reflected in the journal system. Specifically, it assesses the extent to which SDG-related research has been incorporated into both established and newly founded journals, focusing on three SDGs that represent the social (SDG 04: Quality Education), economic (SDG 08: Decent Work and Economic Growth), and environmental (SDG 13: Climate Action) pillars of sustainability. While the 17 SDGs are inherently interconnected and often cluster around shared thematic areas (for example, SDGs 6, 12, 13, 14, and 15 form an ecological grouping), a comprehensive retrieval of SDG-related publications (see Methods) yields millions of articles and associated journal records, making full coverage analytically impractical. Selecting these three SDGs therefore provides a manageable yet analytically meaningful entry point, enabling comparison across domains that differ in disciplinary organization.

### 1.1. A proposed ‘directive shift’ framework

Research has extensively examined how funding agencies, policy instruments, and institutional governance shape the trajectories of scientific fields [24–31]. While the SDGs may be mediated through these mechanisms, for example, by being incorporated into funding calls, institutional strategies, or evaluation criteria, the emphasis here is different. Our focus is on the goals themselves as globally articulated priorities. In this sense, the SDGs represent a “directive shift”: a globally legitimized orientation that guides, signals, and impels research attention toward goals without formally prescribing compliance. As this concept is not established in the sociology or philosophy of science, it is clarified through comparison with other externally driven forms of research organization.

One comparison concerns a crisis-driven mobilization, such as the surge in research during the COVID-19 pandemic [32–35]. Both the Sustainability Development Goals (SDGs) and COVID-19 research illustrate how external pressures can disrupt what Kuhn [2] termed normal science. Yet they differ in temporality and intent: the pandemic produced a reactive, crisis-driven mobilization of research priorities, whereas the SDGs embody a strategic, long-term framework aimed at embedding sustainability as a lasting imperative. This contrast highlights the distinction between temporary disruptions and enduring, directional shifts in the organization of research.

A further comparison can be made between the SDGs and the “War on Cancer,” which, unlike the COVID pandemic, represents a sustained policy-driven mobilization. Initiated through the U.S. National Cancer Act of 1971, this campaign marked a turning point in federal support for cancer research and control [36]. Nevertheless, cancer research has remained episodic, rising and falling with political cycles and shifts in public and governmental attention [37]. As a predominantly top-down initiative rooted in national health agendas, it requires continual renewal to maintain momentum. Despite progress, cancer remains difficult to prevent or treat and continues to be a major global cause of morbidity and mortality [38]. The SDGs likewise demand sustained commitment but differ in scope, extending beyond a single health priority to encompass a far broader range of disciplines and societal goals.

Looking beyond the health domain, the SDGs can also be compared to the U.S. National Nanotechnology Initiative (NNI) and the Human Genome Project (HGP). The NNI exemplifies a coordinated government effort to shape a new scientific and industrial frontier, reorienting national research priorities. The HGP represents a prototypical Grand Challenge: a mission-driven enterprise with a clearly defined objective, i.e., the sequencing of the human genome. It mobilized large-scale international collaboration, funding, and technological innovation, creating new infrastructures and disciplines [39]. By its completion in 2003, it had transformed biology, giving rise to genomics, bioinformatics, and precision medicine [40]. Yet unlike these domain-specific initiatives, the SDGs operate across the entire research landscape.

In broad terms, the sustained goal-oriented research that the SDGs represent is captured by [41,42] Mode 2 framework of knowledge production. Unlike Mode 1 research, which is guided by academic curiosity and discipline-based paradigms, Mode 2 is often guided by external priorities, making it more responsive to societal problems rather than traditional internal ones. Because of the framework, a decline of the disciplinary organization of research was anticipated, though this prediction did not prove to be empirically accurate [43]. Hessels and Van Lente [43] argued that Mode 2 tends to overstate the novelty of contemporary research practices, as many of its defining features were already evident in earlier modes of knowledge production. Their critique cautions against frameworks that risk privileging measurable outputs and performative alignment with societal goals over epistemic depth and exploratory inquiry.

Like Mode 2, the SDGs rely on indicators and reporting mechanisms to assess progress [ 44–47] . SDG language is thus increasingly embedded in funding calls, evaluation criteria, and institutional strategies. Yet sustainability research is not reducible to funding incentives alone. Many SDG themes, such as inequality, labour precarity, and educational access, predate the 2015 Agenda 2030 [48], though their formal codification has strengthened the convergence between normative commitments and institutional incentives. As a result, SDG alignment increasingly shapes how research is framed and positioned, without reducing engagement to purely instrumental motives.

While these comparisons illuminate the SDGs as part of a broader lineage of agenda-driven and mission-oriented research, there are still some reasons why they differ. Unlike initiatives such as the HGP, NNI, or Mode 2-style governance frameworks, the SDGs operate at an unparalleled scale of ambition and scope of influence. **Table 1** below provides a comparative summary of the SDGs and the other research governance models (see **Table 1**).

**Table 1:** Directive Shift Framework Compared to Related Research Governance Models.

Dimension	Funding/ Policy Steering	COVID-19 Surge	War on Cancer	NNI/ HGP (Mission-Oriented)	Mode 2	Directive Shift (SDGs)
**Driver**	Funding agencies and institutional programs	Global health crisis	National health legislation	Government-coordinated strategic agenda	Societal problem orientation	Globally codified normative goals (UN SDGs)
**Scale**	National or institutional	Global but crisis-specific	Primarily national	National or domain-specific international	Variable	Universal (adopted by 193 UN member states)
**Institutional Mediation**	Direct steering via funding and evaluation criteria	Emergency funding reallocations	Federal mandates and funding	Coordinated funding and infrastructure-building	External stakeholder engagement	Mediated through funding and evaluation systems but not reducible to them
**Temporality**	Programmatic and often cyclical	Short-term, reactive	Long-term but politically episodic	Sustained but mission-bound	Ongoing problem responsiveness	Long-term (2030 Agenda), future-oriented
**Scope of Influence**	Field- or program-specific	Primarily biomedical fields	Health domain	Specific technological or scientific domains	Cross-disciplinary but context-dependent	Cross-sectoral and cross-disciplinary (social, economic, environmental)
**Multi-sector Influence**	Mainly academia and policy bodies	Primarily scientific community	Health sector	Research and industry sectors	Academia and societal actors	Governments, academia, business, NGOs, civil society

Note from **Table 1** that a key distinguishing feature of the SDGs is their principle of universal adoption. Again, Kuhn’s [2] account of paradigm shifts suggests that change within science is often gradual and internally contested before becoming widely accepted. In retrospect, the transition may appear sudden, but in practice, it often takes time for a new paradigm to gain widespread acceptance. Because the SDGs were adopted by all 193 United Nations member states in 2015, they possess a unique level of global legitimacy and adoption. Not only does this differ from an internal paradigm shift (with its emphasis on internal ‘crises’), but it is also rare amongst most other external socially embedded directives.

Another significant reason for proposing “directive shift” as a distinct framework relates to the SDGs’ broad, multi-sectoral influence. Unlike national or regional policies, the SDGs extend beyond governmental agendas to shape the priorities of businesses, non-profits, academia, and civil society worldwide [49,50]. Within academia alone, the SDG targets intersect with an unusually wide range of discipline, from environmental sciences and economics to health, education, and the social sciences.

## 2. Background literature

The following sections examine the evolution of sustainability research, ongoing debates regarding its impact, and its progression toward institutionalisation as a field.

### 2.1. Mapping SDG research

The term sustainability became increasingly prevalent in published research from around 1990, with a sharp rise after 2005 [51]. Early efforts to map the structure of sustainability research, using citation networks, revealed a field undergoing rapid integration. A topological clustering of publications from 2007 (n = 29,391) and 2013 (n = 89,908) showed that what had been 15 distinct research clusters in 2007 consolidated into 10 by 2013. The seven largest clusters, covering Environmental Systems, Economy and Business Systems, Fishery and Forestry Systems, Energy Systems, Water Resources, Health, and Urban and Transport Systems, accounted for more than 80% of the literature (p. 425).

Following the introduction of the United Nations Sustainable Development Goals (SDGs), interest in mapping sustainability research expanded considerably (e.g.,[52–62]. Some studies have sought to quantify research contributions to individual SDGs (e.g., [59; 63] or to subsets of goals to distinguish between direct and indirect contributions to the sustainability agenda (e.g., [53]). Others have focused on institutional-level outputs [64] or examined the influence of the SDGs within specific thematic domains [54,63]. Further contributions have identified dominant SDG-related research themes [61,65] as well as leading national contributors [52]. Recently, Confrara et al. (2024) [22] found that countries with “higher SDG challenges are relatively specialised in research related to [the sustainability] goals” (p. 1). This “positive alignment between the countries’ research priorities and SDG challenges”, specifically occurred for “SDG1 (No poverty), SDG2 (Zero hunger), SDG6 (Clean water and sanitation), and SDG9 (Industry, innovation, and infrastructure) (p. 1).

As this body of work expanded, methodological inconsistencies emerged. Armitage et al.‘s [53] solution was to create two independent query constructions, referred to as the Bergen action-approach (BAA) and the Bergen topic-approach (BTA), for retrieving SDG-related publications. The BAA combines topic terms with action-oriented language derived from SDG targets to identify publications that directly address the achievement of specific goals, whereas the BTA removes most action terms and captures research related more broadly to SDG themes. When compared with Elsevier’s proprietary classifications in SciVal, both approaches revealed substantial variation, with particularly limited overlap observed for the more restrictive action-approach.

Subsequent methodological developments sought to improve retrieval precision. Bordignon [66], for instance, demonstrated how the CorTexT text-mining tool could extract domain-specific phrases to enhance database queries. More recently, Lancho Barrantes [62] compared three major platforms, i.e., *SciVal* (Elsevier), *InCites* (Clarivate), and *Dimensions*, and found that *SciVal* indexed the largest number of SDG publications, followed by Dimensions and then *InCites.* To promote greater consistency, standardized search strings have now been developed for all 17 SDGs across major platforms, including *Scopus* [67,68], *InCites* [69], and *OpenAlex*. Nevertheless, results remain sensitive to the choice of database, analytical context, and level of analysis (institutional versus global), as emphasized by Lancho Barrantes [62].

In response to these challenges, alternative approaches have emerged. Ottaviani and Stahlschmidt [70] developed a fine-tuned Large Language Model (LLM) model to improve SDG retrieval, generating a joint SDG publication dataset for three indexes (*Web of Science, Open Alex*, and *Scopus*), comprised of 15,471,336 records (2015–July 2023). For communities without access to commercial databases, Yin et al. [71] illustrate the potential of AI-driven similarity measures and demonstrate how to use AI for large-scale SDG research classification and mapping.

### 2.2. SDG research impact and institutionalisation

Whilst the volume of SDG-related publications appears to be increasing and mapping techniques are improving; questions remain regarding their impact and the extent to which sustainability research is becoming institutionalised as a field.

The origins of sustainability science can be traced to the 1980s, when its primary aim was to understand interactions between society and the environment and to explore pathways toward more sustainable futures [72]. Building on this foundation, Kajikawa [73] outlined key components of the research area, including “goal setting, indicator setting, indicator measurement, causal chain analysis, forecasting, backcasting, and problem–solution chain analysis” (p. 215).

A strong emphasis on problem-solving initially shaped the field’s development. Weik et al. [74] and Yarime et al. [75] emphasized the importance of stakeholder partnerships and integration across education, research, and societal engagement in addressing sustainability challenges. This gave rise to what Schoolman et al. [76] termed the “tripartite” model of sustainability, combining equitable economic growth, social well-being, and thriving natural systems.

Despite these advances, more research is needed to reflect Schoolman et al.‘s [76] integrated model, and to demonstrate real world impacts in practice. Chapman et al. [77] contend that metric assessments of impacts are limited and risk becoming overly “self-referential,” potentially diverting attention from real societal issues. Using a World Café methodology with 51 conference participants, they proposed a new framework. This included: (a) identifying research end-users (students, industry, government, civil society); (b) incorporating end-user evaluations of outcomes; (c) developing shared language between academics and stakeholders; and (d) fostering collaboration across sectors.

According to Sorooshian [78], advancements towards achieving impact have been insufficient, in part because some SDG goals tend to be more research-friendly (i.e., SDG 3-health and well-being) than others (i.e., SDG 4-quality education and SDG 16-peace, justice, and strong institutions). New empirical work further complicates this picture. Biermann et al. (2025) [49], analysing approximately 3,000 SDG studies, found that most impacts to date have been discursive. Instances of normative impact (i.e., changes in laws, regulations, or policies) as well as institutional impact (i.e., the creation or reform of organisations or processes) were less common, while cases of transformational impact—where discursive, normative, and institutional changes converge—were notably rare.

Assessing the institutionalisation of sustainability research has been an equally complex challenge. Earlier, Clark and Dickson [79] found that sustainability research was not yet a consolidated field, rather a problem-driven programme addressing a broad array of fundamental questions. Nonetheless, there was at least a clear impetus toward integration across disciplinary boundaries. Schoolman et al. [76] examined the interdisciplinarity of sustainability research and found uneven integration across the three “pillars” of sustainability, i.e., economic, social, and environmental, observing that economics was comparatively integrative, while environmental sciences remained more self-contained. Their findings also indicated that relatively few interdisciplinary journals had emerged within certain domains, particularly economics and the social sciences.

In a more recent study, Vanhulst et al. [80] suggest that sustainability science has achieved significant interdisciplinary visibility “in terms of publications and institutional relevance,” yet continues to face “substantial epistemological and methodological challenges”. Drawing on sociology of science perspectives (e.g., [3,4,29,81,82], the authors examined how knowledge production practices and epistemic foundations shape the consolidation of sustainability science as a field. Central to this process is reflexivity, that is, scientists’ critical reflection on both what they know and how they know it. Their analysis identified four key dimensions.

The first is the distinction between a science of sustainability (focused on understanding human–environment interactions) and a science for sustainability (oriented toward action). The second concerns the challenge of integrating natural, social, and non-academic knowledge, often complicated by disciplinary hierarchies, methodological differences, and difficulties in validating local knowledge. The third is a public dimension, in which sustainability positions itself as a form of “public science” aimed at societal transformation, though participation may be constrained by tokenism, power asymmetries, and exclusion. Finally, institutionalisation itself plays a dual role: while education and training programmes can embed sustainability across disciplines, they may also introduce rigidity and limit pluralism.

Yarime et al. [75] were the first to suggest that sustainability research would gain legitimacy through the development of dedicated educational programmes, professional associations, specialised journals, and authoritative textbooks. The establishment of the journal *Sustainability Science* represents an early step in this process [83], reflecting both the global scope of the SDGs and a broad range of contribution types, including original articles, reviews, technical reports, and case studies. Extending this perspective, Leoncini et al. [84] analysed the relationship between sustainability research and teaching using a longitudinal dataset (2016–2023) from an Italian university, complemented by 34 years of historical data. Findings revealed an uneven pattern of adoption: while many academics engage in either teaching or research on sustainability (but not both), sustainability teaching has expanded rapidly in recent years, whereas research adoption has followed a slower, more gradual trajectory.

The present study builds on this literature by examining the institutionalisation of sustainability research through academic journals, both newly established and existing. As Parthey [85,86] argues, “research and the necessary freedom of research only arise with its institutionalisation.” Historically, journals have functioned as key infrastructures for disciplinary formation. However, their role has come under increasing scrutiny in light of developments such as open access, impact assessment regimes, flipped publishing models, and predatory publishing [87–94].

Despite these critiques, journals continue to play a central role in shaping research communities. As Neylon [95] suggests, they remain a “strand that links us to the nineteenth century,” providing spaces where communities define boundaries, validate membership, and disseminate knowledge. Their continued relevance depends on how effectively they function as collective goods, enabling shared understanding while supporting the production and circulation of knowledge. Against this backdrop, it becomes particularly meaningful to examine the role of journals at the frontier of SDG-related research.

## 3. Method

### 3.1. Data retrieval and research design

Elsevier’s SDG-specific search queries for SDG 13, SDG 04, and SDG 08 [67] were used to retrieve three distinct sets of Scopus-indexed journal articles published between 1990 and 2024. The full query strategies are extensive and not reproduced here; though, they are publicly available through the Elsevier Data Repository [Elsevier, 2023], ensuring transparency and replicability.

Scopus was selected as the primary data source due to its broad journal coverage across the natural sciences, social sciences, and interdisciplinary domains. This breadth is essential for capturing sustainability-related research spanning social, economic, and environmental pillars. In addition, Scopus provides stable journal identifiers and consistent longitudinal indexing, making it suitable for journal-level analysis over an extended time.

Application of Elsevier’s [96] SDG queries yielded a substantial journal corpus (February 2025). **Table 2** presents the query results for the three selected SDGs, including article and journal counts, the number of journals shared between each SDG area, and the number and percentage of articles with acknowledged funding. Whilst a higher proportion of research corresponding to SDG 13 appears to be associated with external funding (~65%), the absence of detailed information linking specific funding calls to acknowledgements precludes verification of the extent to which sponsoring agencies directly influenced the observed research outputs.

**Table 2:** Elsevier’s SDG query results for SDG04 (Quality Education), SDG08(Decent Work and Economic Growth) and SDG13 (Climate Action).

Sustainable Development Goal	# of articles	# and % of articles with acknowledged funding
SDG 04: Quality Education	431,301	58,796 (~ 13.6%)
SDG08: Decent Work and Economic Growth	597,705	178,411(~ 29.9%)
SDG13: Climate Action	557,232	362,516 (~ 65.1%)
**TOTAL**	**1,586,238**	**599,723 (~ 38%)**

**Fig 1:**
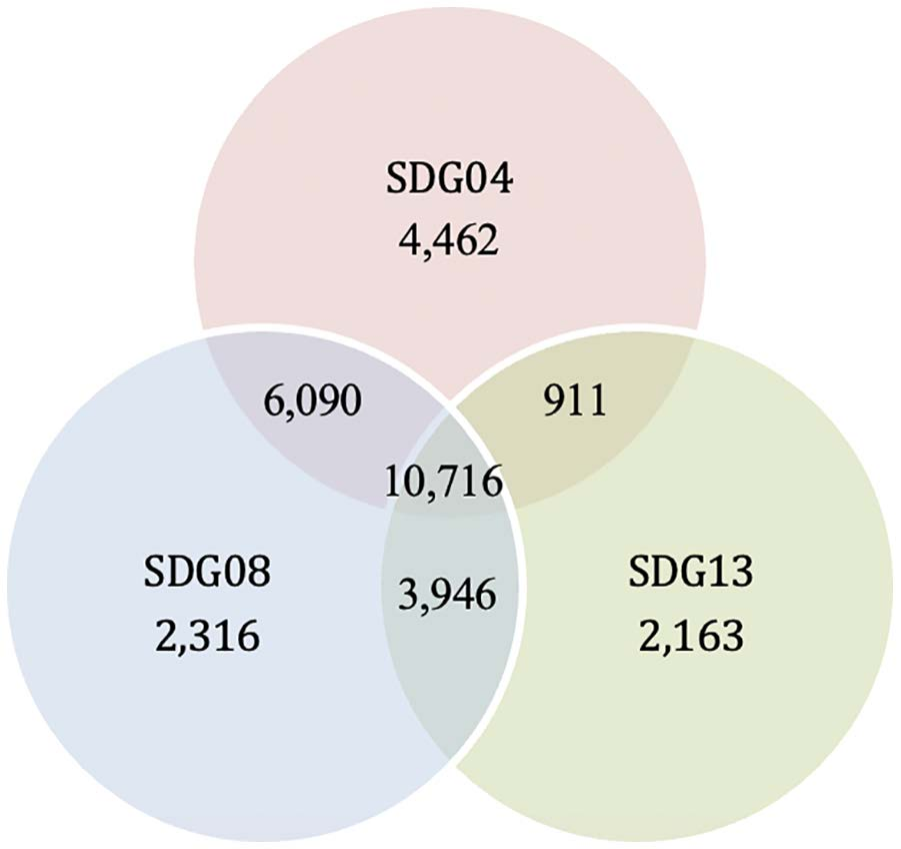
Overlap between publications related to SDG04, SDG08, and SDG13. Venn diagram showing the number of publications associated with Sustainable Development Goals SDG04 (Quality Education), SDG08 (Decent Work and Economic Growth), and SDG13 (Climate Action). Numbers indicate the count of publications unique to each SDG as well as those shared between two or all three SDGs. The largest overlap occurs between all three SDGs (*n* = 10,716), while pairwise overlaps include SDG04–SDG08 (*n* = 6,090), SDG04–SDG13 (*n* = 911), and SDG08–SDG13 (*n* = 3,946).

Whilst funding can and does play an important mediating role, this study does not seek to disentangle funding effects from SDG alignment at the level of individual projects. Instead, it examines whether the SDGs are reflected in the structure and dynamics of the journal system. Any attempt to attribute observed publishing patterns directly to funding would be speculative; therefore, the focus remains on the imprint, or direct influence, of the SDGs themselves (Fig 1).

Given that there is also a clear variation across SDG retrieval approaches and databases [e.g., [53,62], this study will not determine the precision of SDG classification at the article level. Instead, the focus is on structural dynamics within the journal system, for which consistency, transparency, and scalability are more critical than marginal differences in recall or precision. Employing publicly documented and widely used SDG queries thus enhances comparability and methodological clarity.

To assess the institutionalisation of sustainability research in relation to journal dynamics, the following research questions guide this study:

RQ1. What is the number of active journals and entrant journals each year across different *k* thresholds for SDG 04, SDG 08, and SDG 13?

RQ2. How do newborn, young, mature, and established entrant journals contribute to each SDG area from 1990 to 2024?

RQ3. What types of entrant journals appear first within SDG 04, SDG 08, and SDG 13: a) single-discipline or multidisciplinary, and b) traditional or open access?

RQ4. What are the survival rates of entrant journals across *k* thresholds for SDG 04, SDG 08, and SDG 13?

### 3.2. Journal classification approach

For each year within the study period (1990–2024), journals were classified as either **active** or **non-active** in a given SDG domain based on the number of relevant articles published in that year (n) relative to a fixed threshold value (*k*). A journal was defined as active if it published at least *k* articles addressing the SDG area under consideration (SDG 04, SDG 08, or SDG 13).

Operationally, journal entry into a given SDG domain is defined as the first year in which it publishes at least *k* articles related to that SDG. By varying the activity threshold (k = 1, 3, 5, 10), an occasional publication is distinguished from a journal with more sustained engagement.

Four different values of *k* (1, 3, 5 and 10) were tested to capture differences in the active journals over time. A threshold of *k* = 1 indicates that a minimum of 1 article (or more) has been published in relation to the SDG goal. At *k* = 3, *k* = 5, and *k* = 10 the threshold is increased to a minimum of 3, 5 and 10 articles respectively. Increasing the value of *k* is expected to yield a more stringent, and potentially more reliable definition of what an active journal is. For instance, a journal might publish a single article in a certain year but focus mainly on topics outside the scope of the specified SDG. While higher thresholds may help to exclude outlets that publish SDG-related papers only sporadically, they also serve to test whether the observed patterns remain stable when focusing on journals whose engagement with the selected SDG is presumably more systematic. Each analysis was conducted twelve times: once for each of the three SDGs and again for each of the four values of the *k* threshold.

Among active journals, two further categories were defined. Journals that had previously met the threshold and continued to do so were classified as **incumbents**. Journals that met the threshold for the first time in year y were classified as **entrants**. This distinction enables analysis of entry dynamics within the journal population over time.

Entrant journals for a given year y were subsequently classified as **newborn**, **young**, **mature**, and **established**, based on their age in that year. Journal age, referred to as “age at SDG entry” in **Table 3**, was calculated using the founding year recorded in Scopus. These categories reflect the life-cycle stage of a journal at the time it first satisfied the SDG activity threshold. Since any fixed cut-off is inherently conventional, a statistical check was carried out to determine if the classifications are robust (see S1 Appendix).

**Table 3:** Classification of entrant journals by age at SDG entry.

Category	Age at Year *y*	Definition	Interpretation at SDG Entry
**Newborn**	0 years (founded in year *y*)	Journals founded in year *y* that publish SDG-related articles in their inaugural year.	Entry into the SDG field occurs at the moment of journal creation.
**Young**	1–10 years	Journals between 1 and 10 years old in year *y*.	SDG engagement begins early in the journal’s life cycle, though not in its founding year.
**Mature**	11–50 years	Journals between 11 and 50 years old in year *y*.	Entry into the SDG field occurs during a mid-life stage of institutional development.
**Established**	More than 50 years	Journals older than 50 years in year *y*.	SDG engagement begins after long-term consolidation and disciplinary stability.

**Fig 2:**
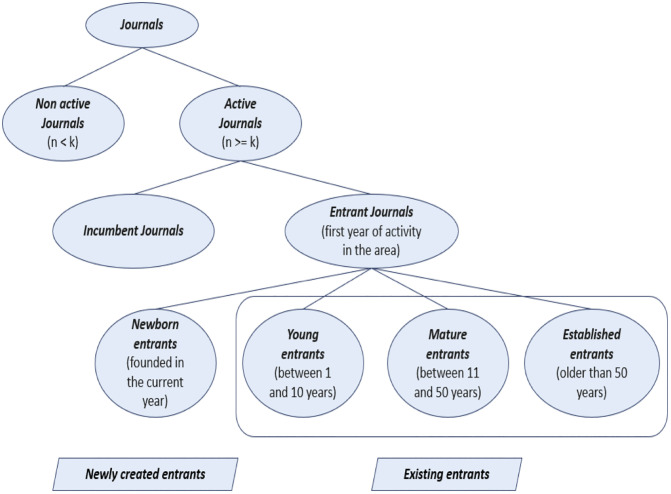
Hierarchy of terms applied to journals (active or non-active) for each SDG-04, 08, and 13. Journal classifications refer to a certain year (y) from 1990 to 2024, with *n* as the number of articles published by the journal in that year, and k represents the fixed value for the threshold.

Fig 2 summarizes the hierarchical classification scheme applied throughout the analysis. Journals are first categorized annually as active or non-active based on the *k* threshold. Active journals are then divided into entrants and incumbents, and entrant journals are further classified by age at the time of SDG entry.

All active journals entering an SDG research area for the first time were further classified according to their degree of multidisciplinary orientation and publishing model, distinguishing between (a) **traditional subscription-based journals** and (b) **open access journals**.

To operationalize disciplinary orientation, journal-level subject classifications were obtained from Scopus. Specifically, a complete list of journals and their associated All Science Journal Classification (ASJC) codes was downloaded from the Scopus source title database [97]. ASJC codes classify journals into one or more subject categories and therefore provide a standardized proxy for disciplinary scope. Journal titles and ISSNs from the SDG 04, SDG 08, and SDG 13 datasets were matched to the Scopus source list using a join procedure in R [98]. Matching was conducted using both journal title and ISSN to ensure consistency and reduce duplication or misclassification. Entrant journals were then classified according to the number of ASJC codes assigned to them. Journals associated with a single ASJC category were categorized as single-discipline journals, reflecting specialization within one primary subject area. Journals assigned two or more ASJC codes were categorized as multidisciplinary journals, indicating broader disciplinary coverage.

To analyse the entry of journals into SDG-related publishing, this study draws on two established traditions that examine entry processes in industrial settings: population ecology [99,100] and industrial organization [101–104]. In both approaches, entry and survival rates are central indicators of structural dynamics.

Applied to scholarly publishing, journals can be understood as organizational actors that enter thematic domains, such as SDG-related research, when they begin allocating editorial attention and publication space to those topics. Entry therefore reflects not only the founding of new journals, but also the reorientation of existing ones toward sustainability themes. Moreover, a journal is defined as exiting when it ceases publishing SDG-related articles (i.e., zero SDG-related output) and does not subsequently re-enter the field during the observation period. For example, a journal that stopped publishing SDG-related articles in 2002 but resumed such publications in 2022 is not classified as having exited. Exit therefore requires a sustained absence through the end of the study period. Entry rates, exit rates, and long-term survival rates are formally defined as follows:

a) The proportion of entrant journals in year *y* relative to the total journals active in the previous year (*y–1*).

$$\begin{array}{c} Entr{y\;rate}_{y}=\frac{{Number\;of\;new\;Entrants}_{y}}{{Total\;Number\;of\;Active\;Journals}_{y-\text{1}}} \end{array}$$

b) The proportion of journals exiting in year *y* relative to the total journals active in the previous year (*y–1*).

$$\begin{array}{c} Exit{\;rate}_{y}=\frac{{Number\;of\;exiting\;journals}_{y}}{{Total\;Number\;of\;Active\;Journals}_{y-\text{1}}} \end{array}$$

c) The share of newly entered journals in year *y* that are still active at the end of the evaluation period.

$$\begin{array}{c} {Survival\;rate}_{y}=\frac{Number\;of\;journals\;still\;active\;at\;end\;of\;evaluation\;period}{{Number\;of\;new\;Entrants}_{y}} \end{array}$$

## 4. Results

The following sections report the empirical findings for SDG 04 (Quality Education), SDG 08 (Decent Work and Economic Growth), and SDG 13 (Climate Action). First, we show both the growth rate and compound annual growth rate of articles for each SDG. Then, starting at section 4.2, the data are used to examine journal entry, activity, age structure, disciplinary orientation, publishing model, and survival across four k thresholds (*k* = 1, 3, 5, 10). These analyses will provide insight into the institutionalisation of sustainability research within the publishing system. All results are presented separately for each SDG to enable comparison across the social, economic, and environmental domains.

### 4.1. Annual growth rate of articles per SDG area

**Fig 3:**
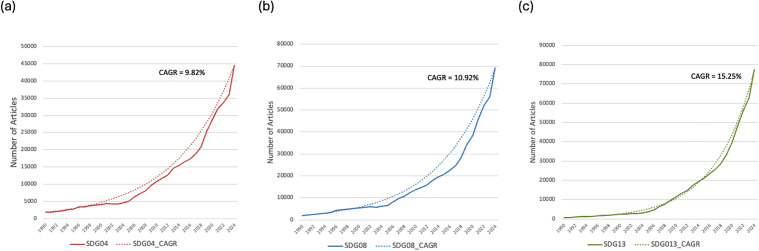
Growth of research articles per SDG area, 1990–2024. (a) Annual growth rate and CAGR for SDG 04 (b) Annual growth rate and CAGR for SDG 08, (c) Annual growth rate and CAGR for SDG 13. Note that the highest CAGR of research articles between 1990 and 2024 corresponds with SDG13 (15.25%), followed by SDG 08 (10.92%) and then SDG04 (9.82%).


Fig 2, illustrates the growth rate as well as the Compound Annual Growth Rate (CAGR) of articles related to each SDG pillar (SDG 04: Quality Education; SDG 08: Decent Work and Economic Growth; SDG 13: Climate Action), with the CAGR calculated as follows (Fig 3):

$$\begin{array}{c} CAGR\;=\;\left[\left(\frac{Ending\;Value}{Beginning\;Value}\right)\frac{\text{1}}{n}-\text{1}\right]\times \;\text{1}\text{0}\text{0} \end{array}$$

### 4.2. Active versus entrant journals

**Fig 4:**
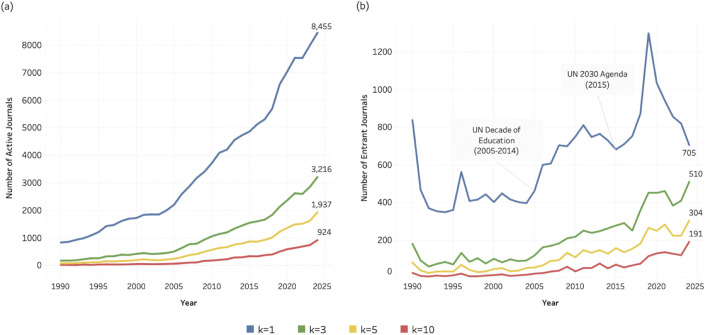
Journal activity and entry patterns for SDG 04 (Quality Education), 1990–2024. (a) Number of active journals per year across k thresholds (k = 1, 3, 5, 10). (b) Number of entrant journals per year across k thresholds (k = 1, 3, 5, 10). Annotations indicate major policy milestones relevant to SDG 04.

**Fig 5:**
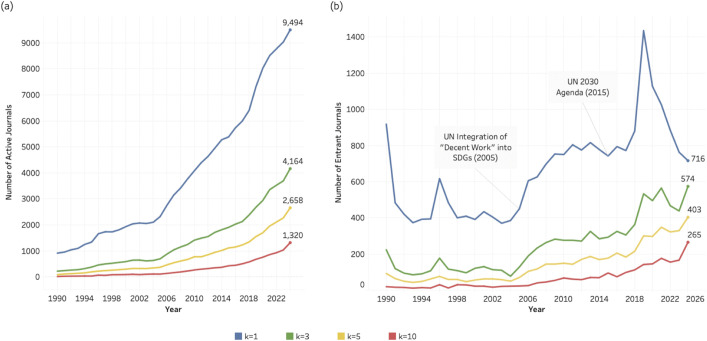
Journal activity and entry patterns for SDG 08 (Decent Work and Economic Growth), 1990–2024. (a) Number of active journals per year across k thresholds (k = 1, 3, 5, 10). (b) Number of entrant journals per year across k thresholds (k = 1, 3, 5, 10). Annotations indicate major policy milestones relevant to SDG 08.

**Fig 6:**
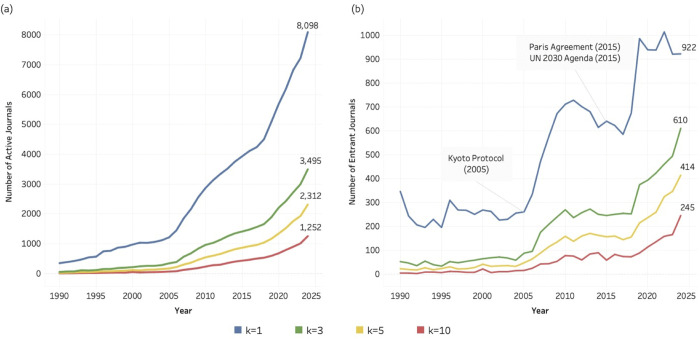
Journal activity and entry patterns for SDG 13 (Climate Action), 1990–2024. (a) Number of active journals per year across k thresholds (k = 1, 3, 5, 10). (b) Number of entrant journals per year across k thresholds (k = 1, 3, 5, 10). Annotations indicate major policy milestones relevant to SDG 13.

Figs 4–6 illustrate the number of journals actively contributing to research related to SDG 04 (Quality Education), SDG 08 (Decent Work and Economic Growth), and SDG 13 (Climate Action) from 1990 to 2024. In each figure, four coloured lines represent the four *k* thresholds (1, 3, 5, and 10). Across all three SDGs, the number of active journals increases steadily over time. At the lowest threshold (*k* = 1, blue lines), two inflection points are particularly noticeable: one around 2005 and another around 2016/2017. At this threshold, defined as journals publishing at least one SDG-related article per year, the number of active journals increases by approximately 2,000 in the final five years of the observation period, exceeding 8,000 across all three SDGs by 2024.

Figs 4b, 5b, and 6b present the time series for entrant journals. At *k* = 1, the entry curves display similar cyclical dynamics across SDGs: a marked rise around 2005, followed by a decline, and a renewed increase around 2015/2016. In Figs 4b and 5b, these shifts coincide temporally with major policy developments, including the UN Decade of Education for Sustainable Development (2005–2014), the 2005 UN World Summit and the formal integration of “Decent Work” into the Millennium Development Goals, and the adoption of the 2030 Agenda in 2015. The entry pattern for SDG 13 (Fig 6b) shows particularly pronounced fluctuations, corresponding to the approval of the Kyoto Protocol (entered into force in 2005) and the Paris Agreement (2015). While these temporal alignments do not establish direct causality, they suggest that international policy milestones may have contributed to observable surges in journal entry. **At** Figs 4b and 5b there are sudden peaks and falls at approximately the year 2019, but only for k = 1 and only for SDG 4 and SDG 8. Here, the minimum entry level of *k* = 1 seems to be related to non-systematic fluctuations, since the pattern is not found for *k* larger than 1.

### 4.3. Age composition *of* entrant journals

**Fig 7:**
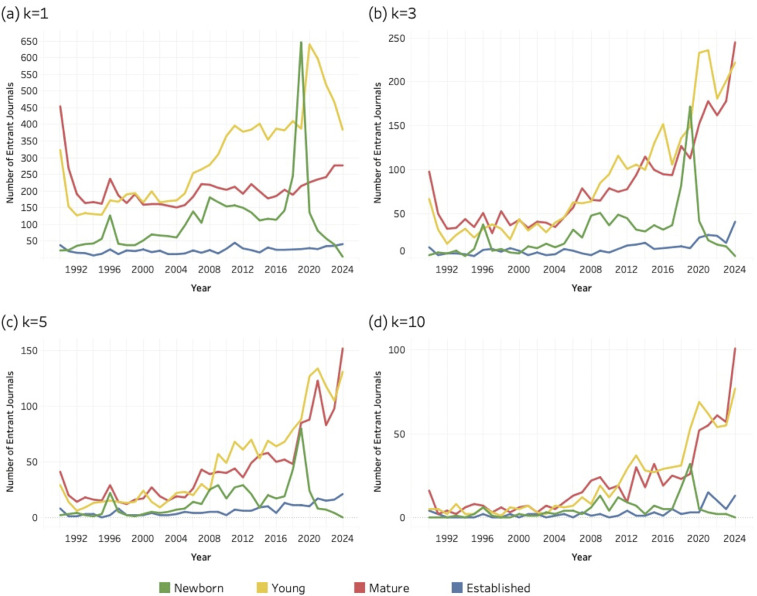
Age distribution of entrant journals for SDG 04 (Quality Education), 1990–2024. (a) k = 1; (b) k = 3; (c) k = 5; (d) k = 10. Lines represent entrant journals classified as newborn, young, mature, or established at the time of SDG entry.

**Fig 8:**
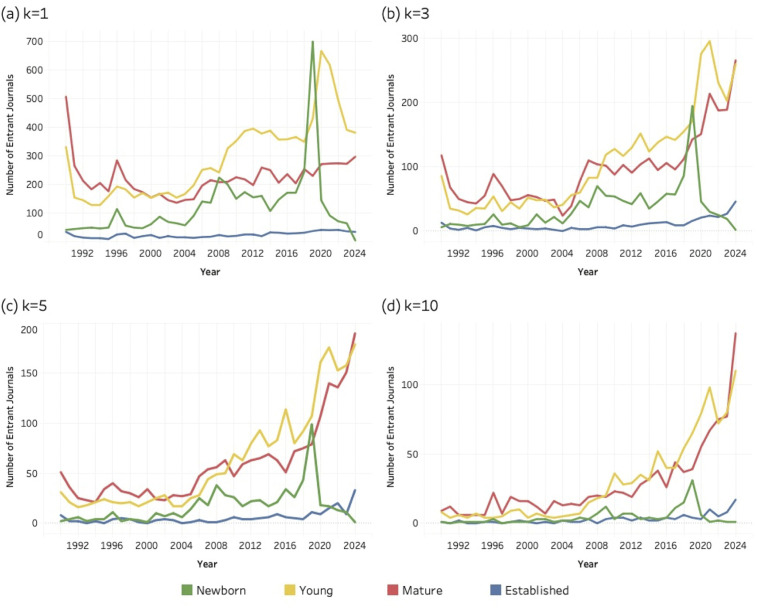
Age distribution of entrant journals for SDG 08 (Decent Work and Economic Growth), 1990–2024. (a) k = 1; (b) k = 3; (c) k = 5; (d) k = 10. Lines represent entrant journals classified as newborn, young, mature, or established at the time of SDG entry.

**Fig 9:**
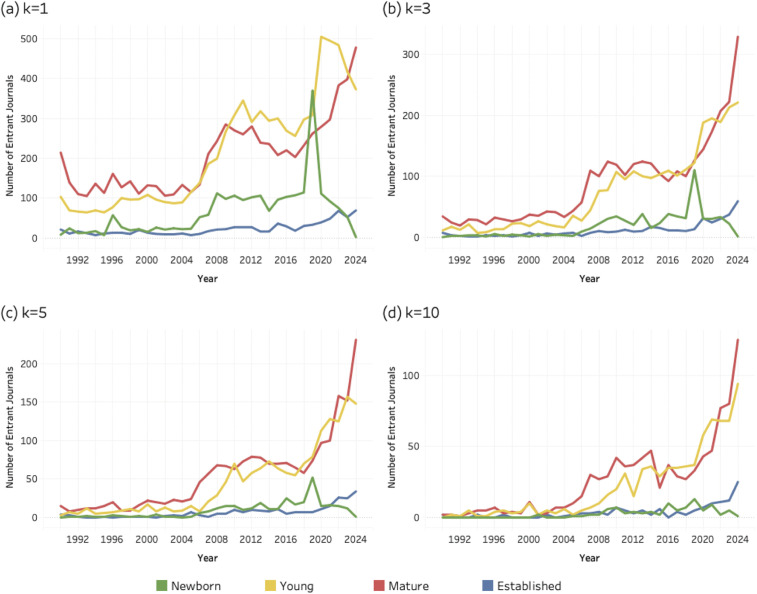
Age distribution of entrant journals for SDG 13 (Climate Action), 1990–2024. (a) k = 1; (b) k = 3; (c) k = 5; (d) k = 10. Lines represent entrant journals classified as newborn, young, mature, or established at the time of SDG entry.

This section compares entrant journals based on their age classes for each of the three sustainability research areas: SDG 04 (Quality Education), SDG 08 (Decent Work and Economic Growth) and SDG 13 (Climate Action). Again, all entrants were classified as newborn (0), young (1–10 years), mature (11–50 years) or established (> 50). Figs 7–9 (below) present a time series for the entrant journals at four different values of the *k* threshold. Coloured lines correspond to each age class (see legend) and the evolving number of entrant journals for each age group.

At Fig 7, for SDG 04 (Quality Education) established journals account for a relatively small share of new global journal entries, showing little growth over time. By comparison, the mature and young journals contribute the most to the population of entrant journals, with their counts systematically exceeding each of the other categories.

When comparing Fig 7 to Fig 8, for SDG 08 (Decent Work and Economic Growth), a similar pattern is found. Established journals are numerically scarce compared to the other categories, mature and young journals consistently exceed the other categories, and newborns account for even lower values than what is observed for the SDG 04 (Quality Education).

At Fig 9, for SDG 13 (Climate Action) the pattern of established journals is again similar to what is shown in the previous two figures. These remain at a very low level and do not grow over time. Moreover, newborn journals contributing to SDG 13 research seem to play a less important role. However, young journals as well as mature journals seem to account for most of the journal entrants.

Newborn journals account for a smaller share of total entrants than young and mature journals. Unlike the broader age cohorts, **newborn** titles are confined to a single founding year, making their presence in the data more sensitive to short-term fluctuations. At the lowest threshold (*k* = 1), in the year 2019, Fig 7a, Fig 8a, and Fig 9a all display a noticeable spike in newborn entrants. These spikes likely reflect the minimal entry requirement at *k* = 1, where the publication of a single SDG-related article is sufficient for inclusion. Newborn journals may incorporate sustainability themes concerning quality education, decent work and economic growth, or climate change into their initial mission statements or inaugural issues or strategically position themselves in relation to emerging policy agendas.

However, as the threshold increases, the number of qualifying newborn journals declines uniformly. This pattern suggests that while some new journals experiment with or signal alignment to SDG topics at the point of entry, fewer sustain that level of engagement over time. Unlike young, mature, and established journals, which have survived academic competition for at least a decade and benefit from reputational legitimacy, newborn journals are subject to the “liability of newness” [105] – i.e., the well-established tendency for newly founded organizations to face higher risks of failure due to limited legitimacy, undeveloped routines, and fragile resource bases. Although originally formulated in 1965, this perspective remains relevant in contemporary scholarly publishing, where new journals must still attract authors and readers, secure indexing in databases such as Scopus or Web of Science and demonstrate financial viability. Their engagement with SDG themes may therefore reflect early positioning efforts rather than durable institutional embedding.

Overall, **established** journals account for a smaller share of entrants than might be expected, a pattern that holds across all three SDGs. This is particularly notable for SDG 08 (Decent Work and Economic Growth), where long-standing journals in economics and management might reasonably be expected to contribute more prominently. The relatively modest presence of **established** outlets suggests that sustainability research has expanded more strongly through younger and mid-life journals than through disciplinary cores. To assess the institutional depth of this pattern more precisely, the following section examines the field position and quartile ranking of established entrants.

### 4.4. Quartile ranking and core-area alignment of established entrants

Although established journals represent the smallest proportion of entrants across all three SDG domains (Figs 7–9), their importance lies in their institutional position rather than their number. Institutionalisation is reflected not only in numerical expansion but in the extent to which new agendas become embedded within long-standing, reputationally consolidated venues. Journals that have survived for more than five decades occupy central positions within disciplinary infrastructures and often function as gatekeepers of scholarly legitimacy [106]. Entry by such journals into SDG-related publishing therefore carries greater institutional weight than entry by newborn or young journals. While newer outlets may respond more readily to emerging policy agendas, established journals typically adopt new thematic orientations more selectively. Their comparatively limited participation suggests that sustainability research has expanded rapidly across the broader journal landscape but has penetrated core disciplinary venues more gradually

To examine this pattern more closely, established entrant journals meeting the most stringent threshold (*k* = 10) were analysed over the period 1990–2020. This higher threshold isolates journals demonstrating sustained engagement with SDG-related publishing. For each SDG, the resulting lists of established entrants are provided in S2 Appendix. The journals listed in here are included because they meet the SDG-specific activity threshold in our dataset *(k* = 10), based on the SDG classification of the articles they publish, rather than because the journals themselves are exclusively associated with a single SDG domain.

For each journal, the SCImago Journal Rank (SJR) quartile at the year of entry was retrieved from Scopus. The SJR indicator classifies journals into four quartiles within their subject field: Q1 (top 25%), Q2 (25th–50th percentile), Q3 (50th–75th percentile), and Q4 (bottom 25%) [107]. Where a journal was not classified within the core subject area of the focal SDG, the quartile was retrieved from its alternative assigned field(s). For years prior to 2000, current quartile rankings were used as a proxy measure of long-term field position.

The distribution of established entrant journals across core subject areas is summarized in **Appendix B, Tables A1** to **A3**. For SDG 04, 39 of the 91 established entrants (fewer than half) are classified within *Education*, the SDG’s core disciplinary field. For SDG 08, 51 of 98 journals (approximately half) are categorized within *Economics or Business Management*. In the case of SDG 13, core classifications are more dispersed, including *Environmental Science* (34 of 127), *Agricultural and Biological Sciences* (20 of 127), *Earth and Planetary Sciences* (5 of 127), *Materials Science* (7 of 127), and *Energy* (4 of 127).

**Fig 10:**
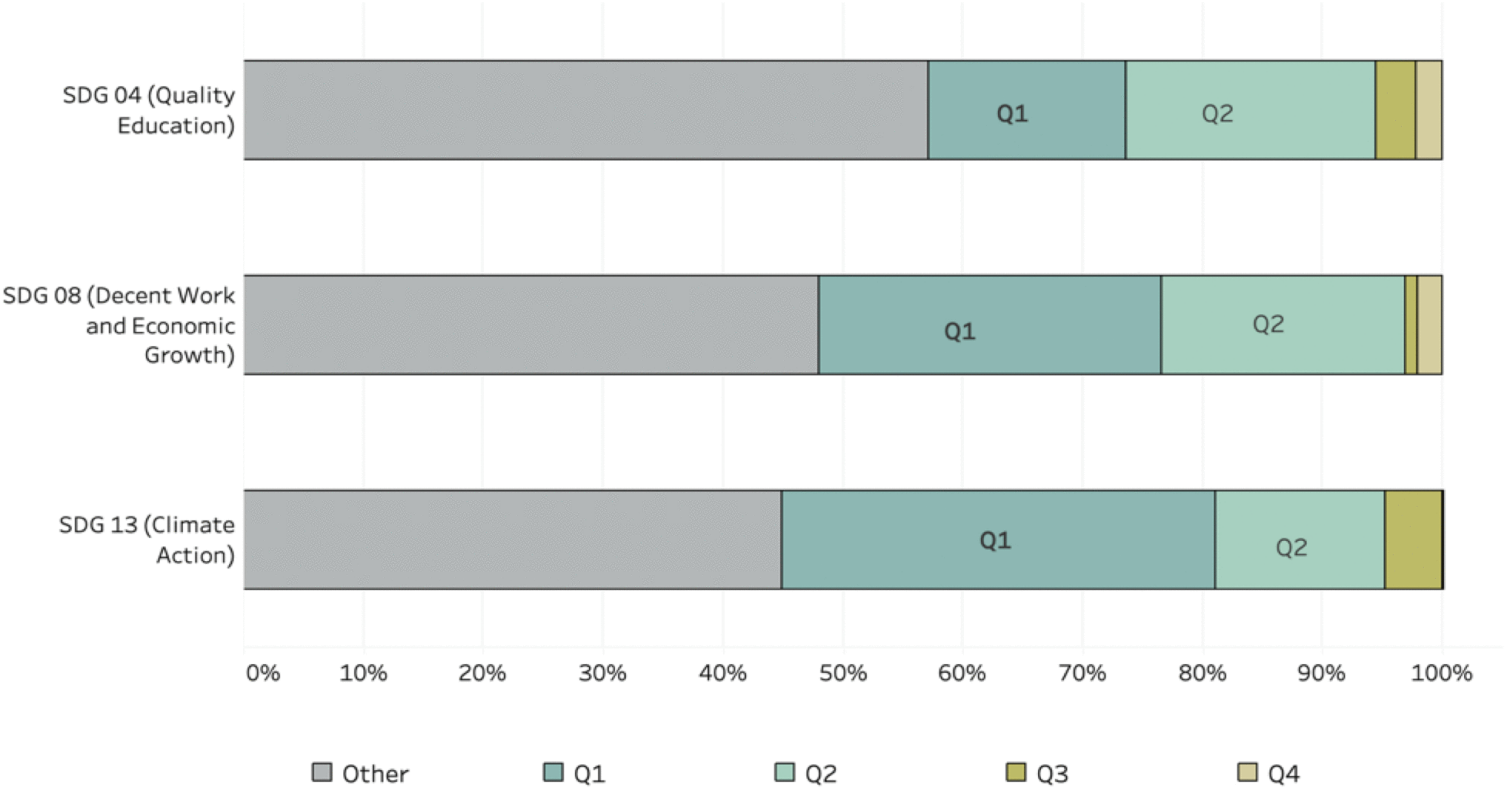
Distribution of established entrant journals by field-related quartile ranking for SDG 04, SDG 08, and SDG 13. Bars represent the percentage of established journals classified as Q1 (top-ranked), Q2, Q3, Q4, or “other,” based on their primary subject category in Scopus.

The bar chart in Fig 10 presents the distribution of established journals according to their field-related quartile rankings. Between approximately 45% and 55% of established journals are classified as “other,” indicating that they are ranked in subject areas not directly aligned with the focal SDG. Among the remaining journals, the smallest share of top-ranked (Q1) established outlets contributes to SDG 04 (Quality Education), followed by SDG 08 (Decent Work and Economic Growth), while SDG 13 (Climate Action) exhibits the largest proportion of Q1-established journal participation.

In the case of SDG 04, core Q1 contributors include established education-focused journals such as *Review of Educational Research*, *Science Education*, and *Educational Technology Research and Development*. For SDG 08, contributions from leading economic and management journals, such as the *Quarterly Journal of Economics*, *Review of Economic Studies*, and *Human Resource Management*, are comparatively limited in proportional terms. By contrast, SDG 13 demonstrates stronger engagement from high-ranking environmental and interdisciplinary outlets, including *Environmental Science, Urban Studies*, and the *Journal of the Science of Food and Agriculture*.

Examples of journals with ‘other’ classifications include *Nature, Proceedings of the National Academy of Sciences (PNAS),* and *Science.* These are the three most prestigious journals (Q1: Multidisciplinary) to have entered sustainability research early within our period of analysis. *Nature* was an established journal entrant in the year 1990 at SDG 13, whilst both *Science* and *PNAS* were established entrants to SDG 13 in the year 1997. It is possible that they entered earlier than 1990, however, since the 1990s they have served as pioneering outlets for *Climate Action* research on a large scale.

The lower percentage of core established (Q1) journal entrants for SDG 04 (Quality Education) and SDG 08 (Decent Work and Economic Growth) may reflect a few different issues. This pattern may be endogenous to the expectations of authors, a journal’s epistemic entrenchment or its degree of methodological conservatism. For example, authors may be dissuaded from submitting articles dealing with economic sustainability to a Q1 economics journal if it has published very little research in the area. Some top economics journals still focus on firm-level efficiency or growth models without integrating sustainability, equity, or well-being measures, thus missing SDG 08’s focus on inclusive and sustainable economic growth. If this is the case, the channelling of sustainability topics to lower-tier journals at Q2 or Q3 signals a problematic dynamic. Another issue could be an established journal’s institutional inertia, or decision to cater to academic prestige rather than applied societal impact. It could also be that many top journals are discipline bound, and not able to become more multidisciplinary as needed to focus on social or economic problem areas.

### 4.5. Disciplinary orientation

**Fig 11:**
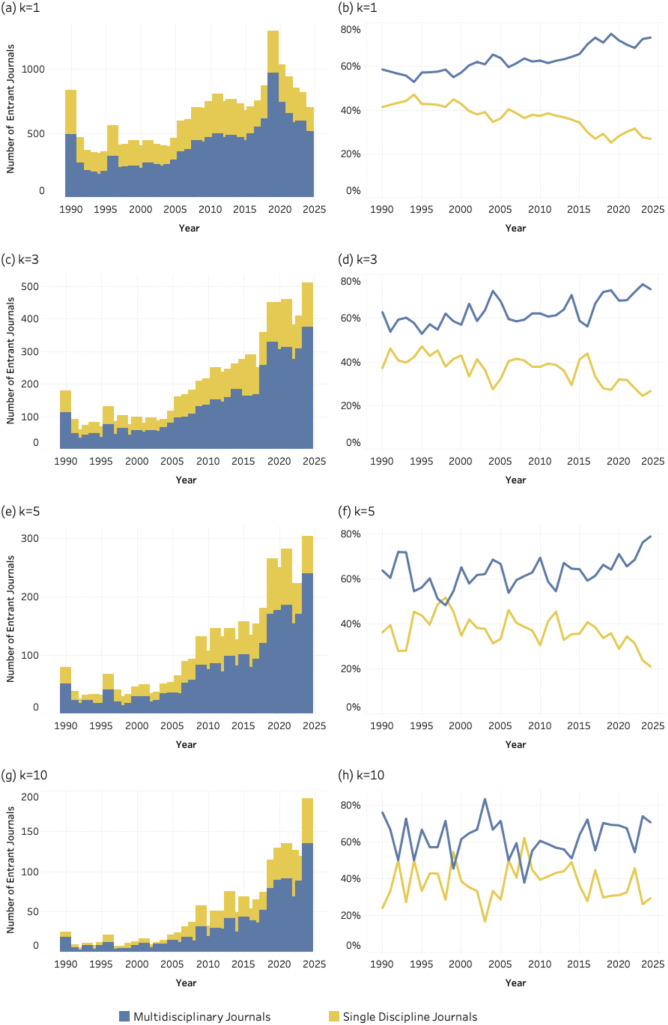
Disciplinary orientation of entrant journals for SDG 04 (Quality Education), 1990–2024. Panels show absolute entrant counts (left column) and percentage share (right column) across k thresholds. Bars represent entrant counts; lines represent percentage share of multidisciplinary and single-discipline journals. (a–b) k = 1; (c–d) k = 3; (e–f) k = 5; (g–h) k = 10.

**Fig 12:**
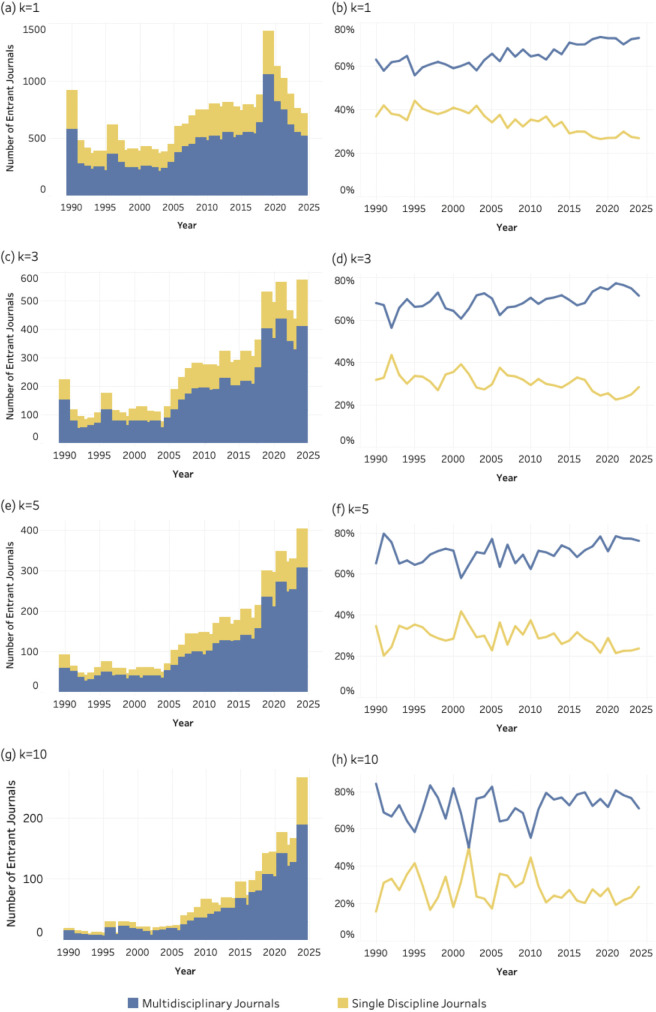
Disciplinary orientation of entrant journals for SDG 08 (Decent Work and Economic Growth), 1990–2024. Panels show absolute entrant counts (left column) and percentage share (right column) across k thresholds. Bars represent entrant counts; lines represent percentage share of multidisciplinary and single-discipline journals. (a–b) k = 1; (c–d) k = 3; (e–f) k = 5; (g–h) k = 10.

**Fig 13:**
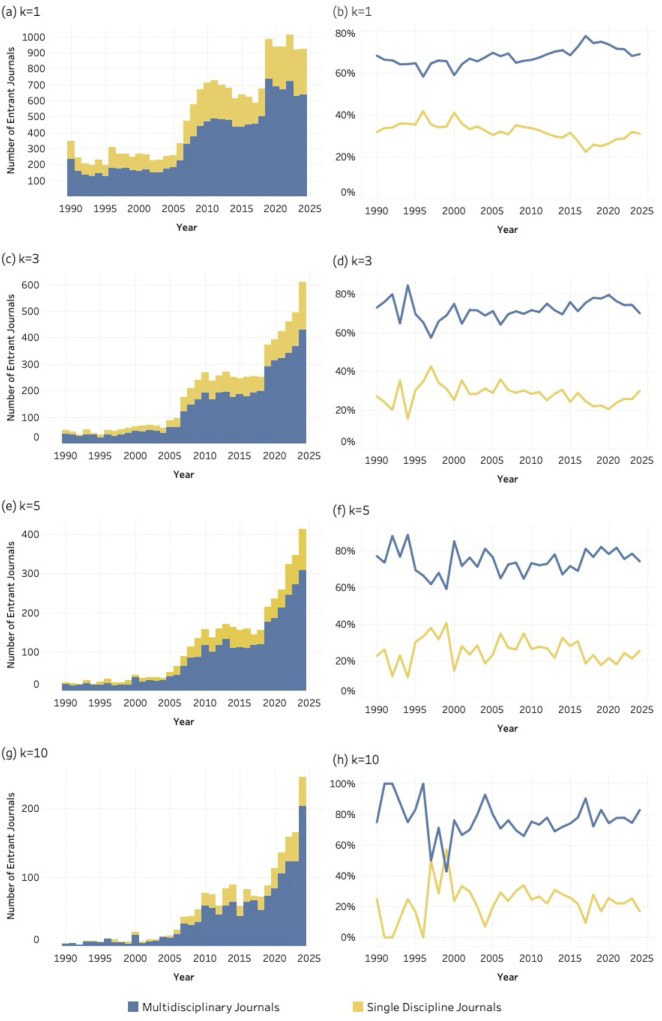
Disciplinary orientation of entrant journals for SDG 08 (Decent Work and Economic Growth), 1990–2024. Panels show absolute entrant counts (left column) and percentage share (right column) across k thresholds. Bars represent entrant counts; lines represent percentage share of multidisciplinary and single-discipline journals. (a–b) k = 1; (c–d) k = 3; (e–f) k = 5; (g–h) k = 10.

The graphs, in Figs 11–13 display both the absolute numbers and proportional shares of multidisciplinary and single-discipline journals for SDG 04 (*Quality Education*), SDG 08 (*Decent Work and Economic Growth*) and SDG 13 (i.e., *Climate Action and Environmental Sustainability*) respectively. Each figure includes eight graphics corresponding to four values of the *k* threshold. At the lowest threshold (*k* = 1), Figs 11 (*Quality Education*) and **12** (*Decent Work and Economic Growth*) reveal a pronounced increase in multidisciplinary entrant journals over time, while the share of single-discipline journals declines steadily. A similar trend is evident for SDG 13 (*Climate Action*), at Fig 13, where multidisciplinary journals dominate across thresholds. This pattern suggests a growing preference for publication venues that integrate multiple disciplinary perspectives.

### 4.6. Publishing model

**Fig 14:**
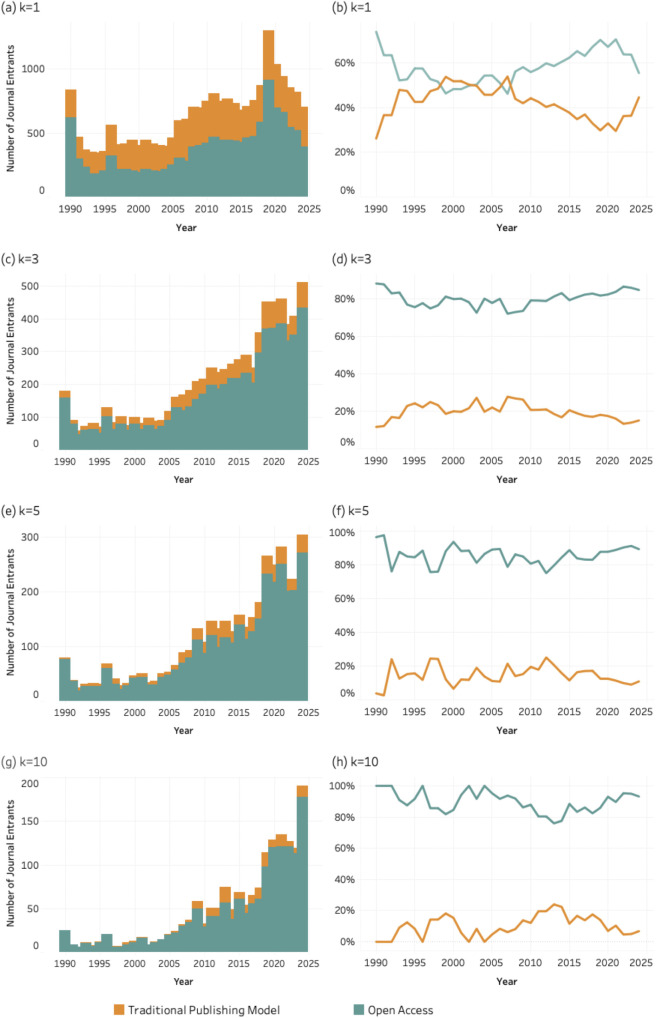
Publishing model of entrant journals for SDG 04 (Quality Education), 1990–2024. Panels display absolute entrant counts (left column) and percentage share (right column) of traditional and open access journals across k thresholds. Bars represent entrant counts; lines represent percentage share by publishing model. (a–b) k = 1; (c–d) k = 3; (e–f) k = 5; (g–h) k = 10.

**Fig 15:**
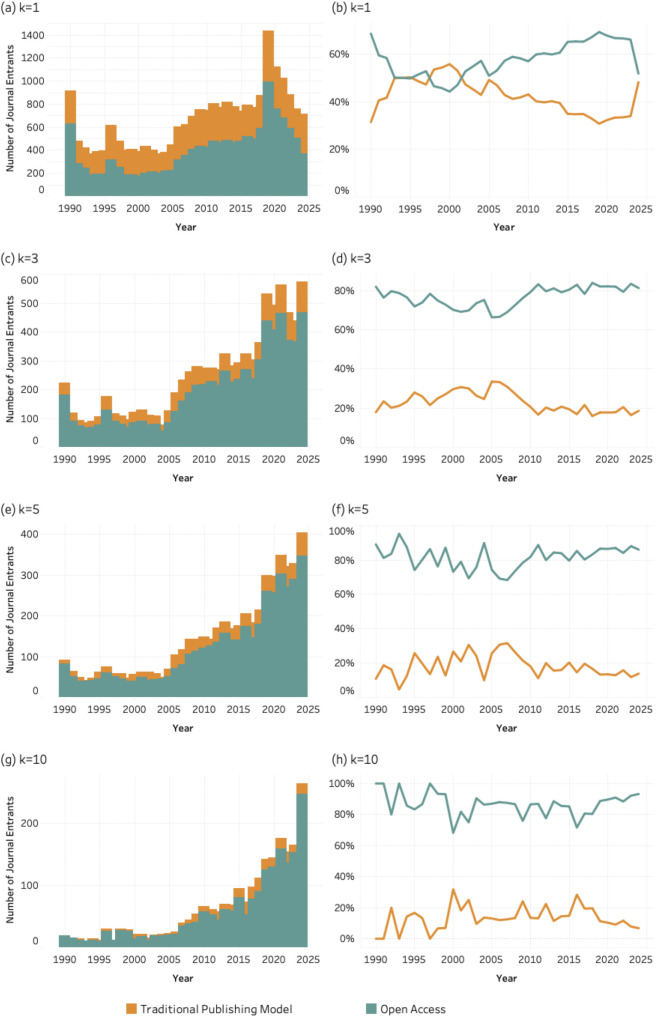
Publishing model of entrant journals for SDG 08 (Decent Work and Economic Growth), 1990–2024. Panels display absolute entrant counts (left column) and percentage share (right column) of traditional and open access journals across k thresholds. Bars represent entrant counts; lines represent percentage share by publishing model. (a–b) k = 1; (c–d) k = 3; (e–f) k = 5; (g–h) k = 10.

**Fig 16:**
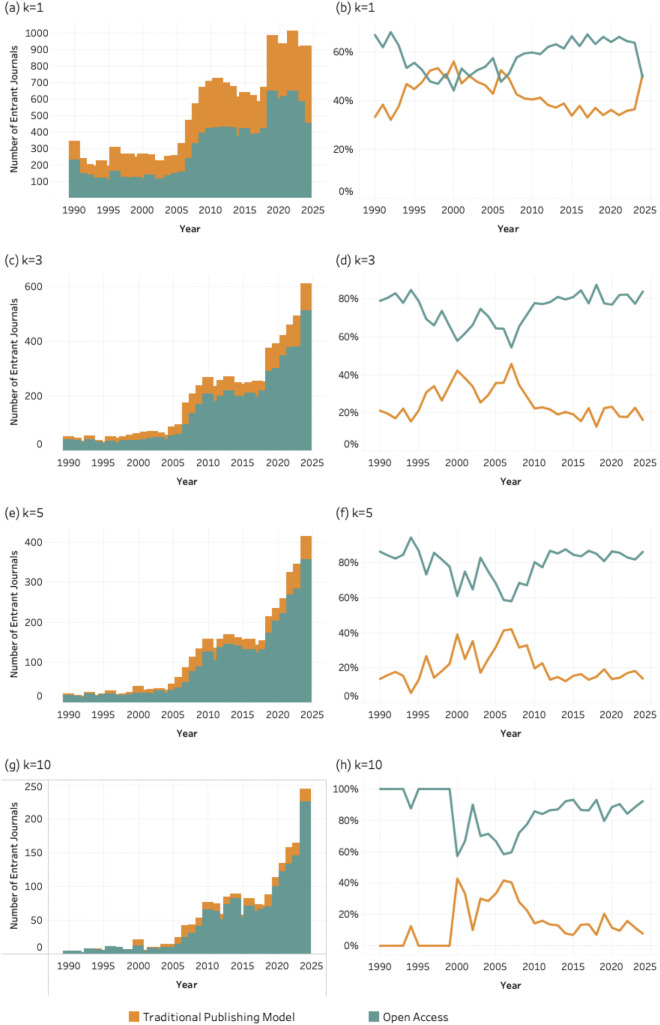
Publishing model of entrant journals for SDG 13 (Climate Action), 1990–2024. Panels display absolute entrant counts (left column) and percentage share (right column) of traditional and open access journals across k thresholds. Bars represent entrant counts; lines represent percentage share by publishing model. (a–b) k = 1; (c–d) k = 3; (e–f) k = 5; (g–h) k = 10.

Figs 14–16 present the number and percentage of entrant journals classified by publishing model (traditional vs. open access) for SDG 04 (*Quality Education*), SDG 08 (*Decent Work and Economic Growth*) and SDG 13 (Climate Action and Environmental Sustainability) across all k thresholds. Each figure includes eight graphics based on the four values of the *k* threshold.

All graphs show that open access journals account for an increasing share of entrants as the *k* threshold rises from 1 to 10. While both publishing models contribute to entry at lower thresholds, open access journals are disproportionately represented among journals meeting higher publication thresholds. The consistency of this trend across social (SDG 04), economic (SDG 08), and environmental (SDG 13) domains suggests that sustained SDG integration is occurring within publishing infrastructures that emphasize accessibility and broader dissemination. In principle, the more open access a journal is, whether newly established as such or converted over time, the more effectively it supports the circulation of key knowledge areas. However, this benefit is not without trade-offs, as open-access models often shift publication costs to authors, introducing new barriers to participation.

### 4.7. Entry–Exit–Survival

**Fig 17:**
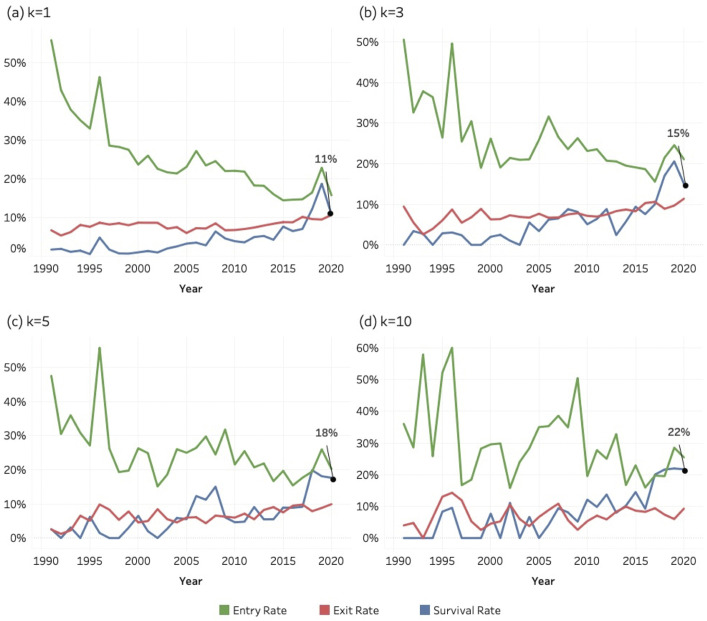
Entry, exit, and survival rates of entrant journals for SDG 04 (Quality Education), 1990–2020. Panels display annual rates calculated relative to the active journal population in year (y–1).(a) k = 1; (b) k = 3; (c) k = 5; (d) k = 10. Lines represent entry rate (green), exit rate (red), and survival rate (blue).

**Fig 18:**
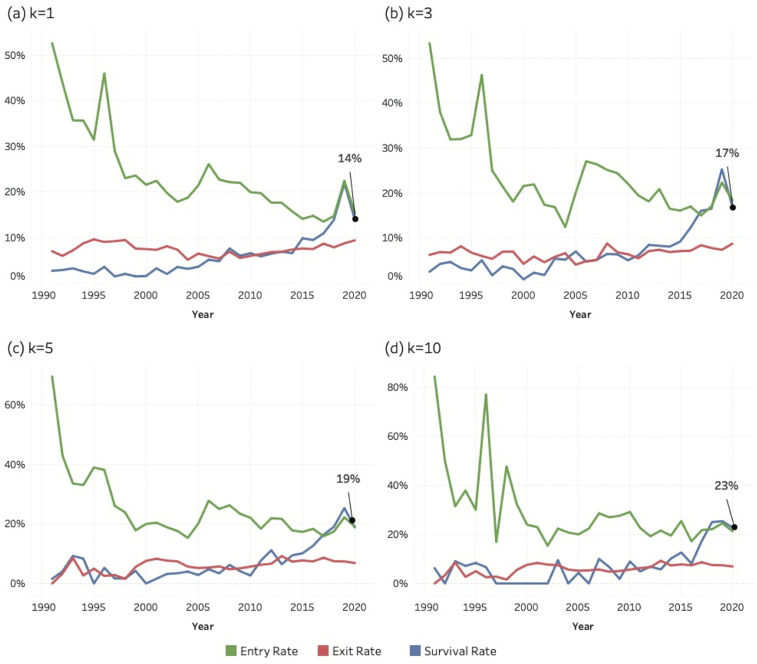
Entry, exit, and survival rates of entrant journals for SDG 08 (Decent Work and Economic Growth), 1990–2020. Panels display annual rates calculated relative to the active journal population in year (y–1). (a) k = 1; (b) k = 3; (c) k = 5; (d) k = 10. Lines represent entry rate (green), exit rate (red), and survival rate (blue).

**Fig 19:**
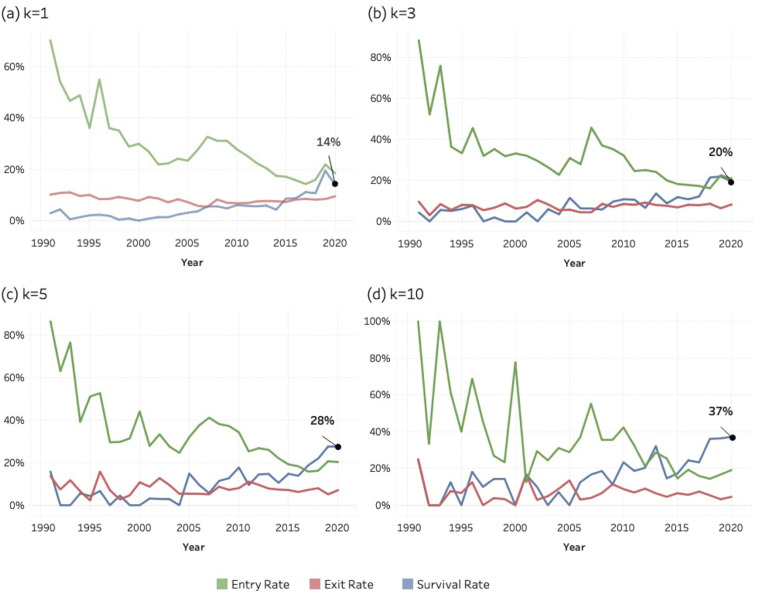
Entry, exit, and survival rates of entrant journals for SDG 13 (Climate Action), 1990–2020. Panels display annual rates calculated relative to the active journal population in year (y–1). (a) k = 1; (b) k = 3; (c) k = 5; (d) k = 10. Lines represent entry rate (green), exit rate (red), and survival rate (blue).

Figs 17–19 show the entry, exit, and survival rate of entrant journals over the period of 1990–2020. These rates are calculated according to the formulas shown in the methods section. The decreasing entry rate (green), which is observable in all the three SDGs and for all the *k* values, reflects a mechanical denominator effect: as the cumulative stock of active journals increases each year, the proportion of entrants necessarily decreases, even though the absolute number of entrant journals entering SDG-related publishing continues to rise. Journals are still continuously entering; hence the entry rate does not decrease to zero.

The analysis concludes in 2020 to allow sufficient follow-up time for assessing exit and survival dynamics. Survival at higher *k* thresholds requires multiple years of sustained publishing activity, and journals entering after 2020 would not yet have had adequate time to meet these criteria. Extending the observation window to more recent years would therefore introduce right-censoring bias and artificially depress observed survival rates. Limiting the analysis to 1990–2020 ensures comparability across cohorts and provides a stable basis for evaluating long-term institutional embedding.

All figures for each of the three SDGs, and at each *k*-threshold show a general increase in survival rate values, especially in the second half of the period. This pattern suggests a growing persistence among journals entering SDG research areas. Higher survival among more recent cohorts indicates that SDG-related publishing is becoming structurally embedded within the scope of journals.

The survival rate for the last year (2020) is indicated as a percentage in each graphic: its value increases systematically at higher k-thresholds across all the three SDGs. Our interpretation of this pattern is that a high threshold of *k* reflects a more stable journal. In other words, a stronger and more regular engagement with SDG topics (i.e., more sustainability-related articles) means that the journal is more likely to survive and become a stable outlet for that SDG area of research. Exit rates remain low and stable across all SDGs, indicating a low hazard of disengagement once journals begin publishing SDG-related content. This pattern supports the interpretation that SDG engagement is persistent rather than episodic. Despite differences in scale—most notably the larger population involved in SDG 13—the structural behaviour of entry, exit, and survival dynamics is remarkably similar across the three SDGs, reflecting the characteristic population dynamics of a consolidating research domain.

While instances of “greenwashing” [108] at the article level cannot be entirely ruled out, patterns of continued publication, particularly at a *k* = 10 threshold, indicate a more genuine institutional engagement. It is improbable that journals consistently publishing sustainability papers per year are engaging in superficial or “greenwashed” academic practices. Conversely, journals that enter the field with only one or two sustainability-related articles per year may be doing so to align with prevailing trends.

## 5. Conclusions

Drawing on bibliometric data covering 30,604 Scopus-indexed journals between 1990 and 2024, this study examined patterns of journal entry, participation, and survival across three sustainability domains. Using activity thresholds ranging from *k* = 1 to *k* = 10 publications, journals were identified that systematically incorporated SDG-related research into their publication portfolios. The results reveal a sustained expansion in journal participation across the three sustainability domains examined. An increasing number of journals enter SDG-related domains over time, and a growing share remain active contributors to publishing in SDG 04 (Quality Education), SDG 08 (Decent Work and Economic Growth), and SDG 13 (Climate Action). The data reveal pronounced surges in journal entry around 2005 and again after 2015, temporally aligning with major international policy milestones.

At the same time, scientific publication output has grown at an average annual rate of approximately 5.08% since 1952, reflecting the long-term expansion of modern science (see [109]). By comparison, the Compound Annual Growth rate (CAGR) of thematic papers was 9.82% for SDG 04, 10.92% for SDG 08, and 15.25% for SDG13 across the 1990–2024 period under examination. Although this exceeds what is known for all publications, it is still important to distinguish this systemic growth from the specific structural consolidation observed here. Our findings do not rest solely on increasing publication volumes, but on patterned entry surges aligned with global policy milestones, the concentration of entrants in multidisciplinary and open-access venues, and rising survival rates at higher publication thresholds. These dynamics indicate institutional embedding rather than uniform background growth.

The patterns observed for disciplinarity, and publishing models further clarify how this consolidation unfolds within the journal system. SDG-related publishing is disproportionately concentrated in multidisciplinary journals and in open-access venues. This suggests that sustainability research is more readily accommodated in publication spaces that are institutionally flexible and oriented toward cross-disciplinary audiences. Multidisciplinary journals provide an organizational platform for integrating perspectives from different scientific domains, while open-access publishing models facilitate broader participation and visibility for research addressing global societal challenges.

In addition, the expansion of SDG-related publishing is supported both by the re-orientation of existing journals, classified as young, mature, or established, and by the appearance of entirely new journals (newborn). Young and mature journals constitute the primary contributors to this process, while newborn and established journals account for a comparatively smaller share. The relatively limited participation of long-standing disciplinary journals, particularly in SDG 04 and SDG 08, suggests that entrenched disciplinary traditions and editorial norms may moderate the pace at which sustainability themes are integrated into established fields.

The quartile analysis further highlights uneven patterns of institutional embedding across disciplines. In the case of SDG 08, journals that systematically publish sustainability-related economic research rarely belong to the most established segment of the field (i.e., journals older than fifty years). Among the highly ranked economics journals, only a small number appear as established entrants, and only two of the so-called “top five” economics journals, i.e., *Quarterly Journal of Economics* (entry in 1992) and *Review of Economic Studies* (entry in 2004) meet the threshold for sustained engagement. Given the centrality of these journals in academic career advancement [110], this pattern suggests that sustainability-oriented economic research remains only partially integrated within the core evaluative structures of mainstream economics.

This disciplinary asymmetry contrasts with SDG 13 (Climate Action), where leading generalist and high-impact journals, such as *Nature, Science*, and *PNAS,* engaged with climate-related sustainability research at an early stage and have supported it consistently over several decades. The comparison indicates that sustainability themes penetrate disciplinary hierarchies unevenly, depending on epistemic traditions, incentive structures, and editorial priorities.

Taken together, these findings suggest that the SDGs operate as an external directive shift in science: a globally legitimized orientation that encourages the institutionalization of sustainability research within the journal system while simultaneously revealing the structural constraints of entrenched disciplinary hierarchies.

### 5.1. Study limitations and future directions

A few limitations of this study point to directions for future research. First, the analysis focuses on three SDGs representing the social, economic, and environmental pillars of sustainability. While this allows structured comparison across relatively unique, yet overlapping disciplinary domains, future work could examine a broader set of SDGs to determine whether similar patterns emerge across other sustainability agendas. Second, our analysis operates at the level of journals rather than individual articles or research networks. Further research could therefore explore collaboration structures, citation dynamics, or topic evolution within SDG-related research. Finally, additional work is needed to examine the mechanisms through which directive shifts occur, including the roles of funding priorities, institutional strategies, and editorial decision-making.

## Supporting information

S1 Appendix Age Classifications Robustness Check. (DOCX)

S2 Appendix Established Journals (k=10). (DOCX)
