# Supplementary material for: The UN SDGs as a global ‘directive shift’ and the institutionalization of sustainability research
Source: PLoS One. 2026 Jun 3;21(6):e0348507. doi: 10.1371/journal.pone.0348507 (PMC13232950; doi:10.1371/journal.pone.0348507)
Supplement: S1 Appendix — (DOCX) [file pone.0348507.s001.docx]

**Appendix A**. Age Classifications Robustness Check

*1. Operationalization of variables*

The classification of active journals into the four age classes - newborn, young, mature and established is based on the age of the journal at the time of entry to the SDG s research area. For each journal j, let F_j_be its founding year and E_j,s_ ​ the year when j first became active in SDG s research (i.e., reached the minimum number of articles on related topics). We define the **age at SDG-entry** as:

$$A_{j,s}= F_{j}- E_{j,s} \in Z$$

Journals are mapped to the classes via the thresholds vector *t (t_1_, t_2_)*:

- *Newborn* if A_j,s_ = 0
- *Young* if $1\leq A_{j,s}\leq t_{1}$
- *Mature* if $t_{1}<A_{j,s}\leq t_{2}$
- *Established* if $A_{j,s}>t_{2}$

The threshold vector only counts two elements (t_1_, boundary between young and mature, and t_2_, between mature and established), because the newborn boundary is definitional (age at entry is 0 by definition). Therefore, we did not perturb it in the robustness check. In the baseline specification we set ($t_{1}, t_{2}$) = (10, 50).

*2. Robustness to threshold jitters (validation test)*

To assess whether findings depend on small, symmetric changes in the cut-offs, we perturbed the thresholds by integer steps $d_{1}, d_{2}\in\{-2, -1, 0, 1, 2\}.$ The words “thresholds” and “cut-offs” are used here with the same meaning.

For each admissible pair we set:

$t' = ({t'}_{1}, {t'}_{2}) = (t_{1}+d_{1},t_{2}+d_{2})$,

subject to ${t'}_{1}\geq1$ and ${t'}_{1}\leq$​ ${t'}_{2}$ (to avoid overlapping intervals).

Let $C_{t}(A_{j,s}) \in\left\{ N, Y, M, E \right\}$ denote the class assigned to j under the original thresholds ($t)$, and $C_{t'}(A_{j,s})$the class under the perturbed thresholds ($t'$). The validation analysis focuses on journals with $A_{j,s} > 0$, i.e., classes young, mature, and established at the time of entry. This set is denoted by $J_{s}^{+}$ and its size by $N_{s}^{+}= |J_{s}^{+}|$ .

Our stability metric is the ***Reclassification Factor* (RF)**, i.e., the fraction of journals that would change class under $t'$ relative to the baseline $t$ (i.e., the journals for which $C_{t}(A_{j,s}) \neq C_{t'}(A_{j,s})$):

$${RF}_{s}(d_{1}, d_{2}) = \frac{1}{N_{s}^{+}} \sum_{j \in J_{s}^{+}} 1\left\{ C_{t}(A_{j,s}) \neq C_{t'}(A_{j,s}) \right\}$$

We computed ${RF}_{s}(d_{1}, d_{2})$ for all admissible combinations $(d_{1}, d_{2})$ with steps $\pm0, 1, 2$ years and summarized its distribution (quartiles, minimum and maximum values).

*Results (quartiles of the RF distribution over all admissible perturbations, for each SDG)*

|  | *Min* | *1^st^ quartile* | *Median* | *3^rd^ quartile* | *Max* |
| --- | --- | --- | --- | --- | --- |
| *SDG 04* | 0.00 | 0.0284 | 0.0343 | 0.0627 | 0.0692 |
| *SDG 08* | 0.00 | 0.0297 | 0.0391 | 0.0562 | 0.0747 |
| *SDG 13* | 0.00 | 0.0240 | 0.0325 | 0.0511 | 0.0687 |

For SDG 04, the first quartile (0.0248) indicates that, in the most favourable 25% of perturbations, fewer than ~2.5% of the journals would change age class; even after the most unfavourable perturbations, reclassifications remain below ~7%. SDG 08 and SDG 13 display similar patterns, supporting the ***robustness*** of the baseline thresholds to ±2-year shifts.
