# Supplementary material for: The UN SDGs as a global ‘directive shift’ and the institutionalization of sustainability research
Source: PLoS One. 2026 Jun 3;21(6):e0348507. doi: 10.1371/journal.pone.0348507 (PMC13232950; doi:10.1371/journal.pone.0348507)
Supplement: S2 Appendix — (DOCX) [file pone.0348507.s002.docx]

$$Entr{y rate}_{y}=\frac{{Number of new Entrants}_{y}}{{Total Number of Active Journals}_{y-1}}$$

$$Exit{rate}_{y}=\frac{{Number of exiting journals}_{y}}{{Total Number of Active Journals}_{y-1}}$$

$${Survival rate}_{y}=\frac{Number of journals still active at end of evaluation period}{{Number of new Entrants}_{y}}$$

$$CAGR = \left[ \left( \frac{Ending Value}{Beginning Value} \right)\frac{1}{n}-1 \right]\times100$$

$$A_{j,s}= F_{j}- E_{j,s} \in Z$$

$1\leq A_{j,s}\leq t_{1}$ $t_{1}<A_{j,s}\leq t_{2}$ $A_{j,s}>t_{2}$ $t_{1}, t_{2}$ $d_{1}, d_{2}\in\{-2, -1, 0, 1, 2\}.$ $t' = ({t'}_{1}, {t'}_{2}) = (t_{1}+d_{1},t_{2}+d_{2})$ ${t'}_{1}\geq1$ ${t'}_{1}\leq$ ${t'}_{2}$ $C_{t}(A_{j,s}) \in\left\{ N, Y, M, E \right\}$ $t)$ $C_{t'}(A_{j,s})$ $t'$ $A_{j,s} > 0$ $J_{s}^{+}$ $N_{s}^{+}= |J_{s}^{+}|$ $t'$ $t$ $C_{t}(A_{j,s}) \neq C_{t'}(A_{j,s})$

$${RF}_{s}(d_{1}, d_{2}) = \frac{1}{N_{s}^{+}} \sum_{j \in J_{s}^{+}} 1\left\{ C_{t}(A_{j,s}) \neq C_{t'}(A_{j,s}) \right\}$$

${RF}_{s}(d_{1}, d_{2})$ $(d_{1}, d_{2})$ $\pm0, 1, 2$**Appendix B**. Established Journals (*k*=10)

The journals listed below are the titles that met the SDG activity threshold (k = 10) in our dataset. Journals titles that are underlined reappear in at least one of the other SDG areas.

**Table 1**. SDG 04 (*Quality Education*)

| **Year** | **Established list** | **Quartile Rank for core relevant areas in SJR** | **Quartiles in other thematic areas (or specific minor categories)** |
| --- | --- | --- | --- |
| 1990 | British Journal of Educational Psychology,  Exceptional Children,  Journal of Educational Psychology,  Journal of Educational Research | Q1* in Education  Q1* in Education  Q1* in Education  Q2* in Education | Other thematic areas: Q1* in Psychology (Educational Psychology)  Specific category: Q1* in Developmental and Educational Psychology  Other thematic areas: Q1* in Psychology (Educational Psychology)  Specific category: Q2* in Educational Research |
| 1991 | Annals of the American Academy of Political and Social Science,  NASSP Bulletin | Not classified in Education  Q3* in Education | Q1* in Social Sciences  Specific category: Q3* in Education |
| 1992 | None |  |  |
| 1993 | None |  |  |
| 1994 | None |  |  |
| 1995 | None |  |  |
| 1996 | Archives of Physical Medicine and Rehabilitation,  JAMA | Not classified in Education  Not classified in Education | Q1* in Medicine  Q1* in Medicine |
| 1997 | None |  |  |
| 1998 | None |  |  |
| 1999 | Jahrbucher fur Nationalokonomie und Statistik,  Pediatrics | Not classified in Education  Not classified in Education | Q2* in Economics, Econometrics and Finance  Q1* in Medicine |
| 2000 | None |  |  |
| 2001 | British Dental Journal,  Educational Review | Not classified in Education  Q2 in Education | Q2 in Dentistry  Specific category: Q2 in Educational Research |
| 2002 | American Annals of the Deaf,  Journal of Dental Education | Q4 in Education  Q2 in Education | Specific categories: Q4 in Developmental and Educational Psychology; Speech and Hearing  Specific categories: Q2 in Dentistry; Education |
| 2003 | None |  |  |
| 2004 | Peabody Journal of Education | Q2 in Education | Specific category: Q2 in Education |
| 2005 | American Journal of Pharmaceutical Education,  British Journal of Educational Studies | Q2 in Education  Q2 in Education | Other thematic areas: Q2 in Pharmacology, Toxicology and Pharmaceutics  Specific category: Q2 in Education |
| 2006 | None |  |  |
| 2007 | Journal of School Health,  Language Learning,  Medical Journal of Australia | Q2 in Education  Q1 in Education  Not classified in Education | Other thematic areas: Q2 in Public Health, Environmental and Occupational Health  Other thematic areas: Q1 in Linguistics and Language  Q1 in Medicine |
| 2008 | American Journal of Surgery | Not classified in Education | Q1 in Medicine |
| 2009 | Review of Educational Research,  Science Education | Q1 in Education  Q1 in Education | Other thematic areas: Q1 in Social Sciences  Specific category: Q1 in Education |
| 2010 | None |  |  |
| 2011 | Journal of Chemical Education | Q1 in Education | Other thematic areas: Q2 in Chemistry |
| 2012 | Australian Occupational Therapy Journal,  Educational Technology Research and Development,  Educational Theory,  Teoriya i Praktika Fizicheskoy Kultury | Not classified in Education  Q1 in Education  Q2 in Education  Q4 in Education | Q1 in Health Professions  Specific category: Q1 in Education  Specific category: Q2 in Education  Specific category: Q4 in Education |
| 2013 | Educational Forum | Q2 in Education | Specific category: Q2 in Education |
| 2014 | Folia Phoniatrica et Logopaedica | Not classified in Education | Q3 in Medicine |
| 2015 | American Journal of Public Health,  Laryngoscope,  Postgraduate Medical Journal | Not classified in Education  Not classified in Education  Not classified in Education | Q1 in Medicine; Social Sciences  Q1 in Medicine  Q2 in Medicine |
| 2016 | Clinical Orthopaedics and Related Research | Not classified in Education | Q1 in Medicine |
| 2017 | Demography,  Health Education Journal,  Journal of Surgical Research,  JPMA. The Journal of the Pakistan Medical Association,  Military Medicine | Not classified in Education  Q2 in Education  Not classified in Education  Not classified in Education  Not classified in Education | Q1 in Social Sciences  Other thematic areas: Q2 in Health Professions  Q1 in Medicine  Q3 in Medicine  Q3 in Medicine |
| 2018 | Quality and Quantity,  Zeitschrift fur Allgemeinmedizin | Not classified in Education  Not classified in Education | Q2 in Social Sciences  Q3 in Medicine |
| 2019 | ACS Symposium Series,  Management Decision,  Proceedings of the National Academy of Sciences of the United States of America | Not classified in Education  Not classified in Education  Not classified in Education | Q4 in Chemical Engineering; Chemistry  Q2 in Business, Management and Accounting  Q1 in Multidisciplinary |
| 2020 | Children,  Journal of Development Studies,  Medicine (United States) | Not classified in Education  Not classified in Education  Not classified in Education | Q2 in Medicine (Pediatrics and Child Health)  Q1 in Social Sciences (Development)  Q3 in Medicine |
| 2021 | Annals of Surgery,  Educational Studies in Mathematics,  Environmental Research,  Higher Education Quarterly,  Indian Journal of Ophthalmology,  International Journal of Electrical Engineering and Education,  Journal of Experimental Education,  Journal of Nursing,  Journal of Paediatrics and Child Health,  Law Teacher,  Neurology,  Physics,  Plastic and Reconstructive Surgery Education,  Voprosy Istorii,  Wiadomosci Lekarskie | Not classified in Education  Q1 in Education  Not classified in Education  Q2 in Education  Not classified in Education  Q2 in Education  Q1 in Education  Not classified in Education  Not classified in Education  Q2 in Education  Not classified in Education  Not classified in Education  Not classified in Education  Not classified in Education  Not classified in Education | Q1 in Medicine  Specific category: Q1 in Education  Q1 in Environmental Science  Specific category: Q2 in Education  Q2 in Medicine  Specific category: Q2 in Education  Specific category: Q1 in Education  Q1 in Nursing  Q2 in Medicine (Pediatrics and Child Health)  Specific category: Q2 in Education  Q1 in Medicine  Q1 in Physics and Astronomy  Q3 in Medicine  Q4 in History  Q4 in Medicine |
| 2022 | American Journal of Tropical Medicine and Hygiene,  American Surgeon,  Australian and New Zealand Journal of Obstetrics and Gynaecology,  British Journal of Social Work,  Indian Journal of Surgery,  Interchange,  Journal of Biological Education,  Journal of Librarianship and Information Science,  Journal of Philosophy of Education,  Physical Therapy | Not classified in Education  Not classified in Education  Not classified in Education  Not classified in Education  Not classified in Education  Q2 in Education  Q2 in Education  Q1 in Education  Q1 in Education  Q1 in Education | Q1 in Medicine  Q3 in Medicine  Q2 in Medicine  Q1 in Social Sciences  Q3 in Medicine  Specific category: Q2 in Education  Specific categories: Q2 in Education; Ecology, Evolution, Behavior and Systematics  Other thematic areas: Q1 in Computer Science; Social Sciences (Library and Information Sciences)  Other thematic areas: Q1 in Philosophy  Other thematica areas: Q1 in Medicine; Health Professions |
| 2023 | Acta Psychologica,  Irish Journal of Medical Science,  Learning and Motivation,  Social Policy and Administration,  Southern Medical Journal | Q3 in Education  Not classified in Education  Q3 in Education  Not classified in Education  Not classified in Education | Other thematic areas: Q2 in Psychology; Neuroscience (Developmental and Educational Psychology)  Q2 in Medicine  Other thematic areas: Q3 in Psychology (Developmental and Educational Psychology)  Q1 in Social Sciences (Development; Public Administration)  Q3 in Medicine |
| 2024 | Acta Medica Philippina,  Child Psychiatry and Human Development,  Economic Journal,  ELT Journal,  IEEE Transactions on Engineering Management,  International Nursing Review,  IRAL - International Review of Applied Linguistics in Language Teaching,  Journal of educational administration and history,  Journal of Moral Education,  Journal of Public Economics,  Public Health Nursing,  RELC Journal,  Socio-Economic Planning Sciences | Not classified in Education  Q2 in Education  Not classified in Education  Q1 in Education  Not classified in Education  Not classified in Education  Q2 in Education  Q2 in Education  Q1 in Education  Not classified in Education  Not classified in Education  Q2 in Education  Not classified in Education | Q3 in Medicine  Other thematic areas: Q2 in Psychology; Medicine (Developmental and Educational Psychology)  Q1 in Economics, Econometrics and Finance  Specific categories: Q1 in Linguistics and Language; Education  Q1 in Business, Management and Accounting; Engineering  Q1 in Nursing  Other thematic areas: Q2 in Linguistics and Language  Other thematic areas: Q2 in History  Other thematic areas: Q1 in Philosphy  Q1 in Economics, Econometrics and Finance  Q2 in Nursing (Public Health, Environmental and Occupational Health)  Other thematic areas: Q2 in Linguistics and Language  Q1 in Social Sciences |
| *: This symbol indicates the current quartile of the journal. We used current quartiles as a proxy for the years before 1999, when the Scimago Journal Rank (SJR) didn't exist.  Core thematic areas relavant to the SDG 04 in the Scimago classification:  Education (Thematic area or Major Subject Category) | | | |

**Table 2.** SDG 08 (*Decent Work and Economic Growth*)

| **Year** | **Established List** | **Quartile Rank for core relevant areas in SJR** | **Quartiles in other thematic areas (or specific minor categories)** |
| --- | --- | --- | --- |
| 1990 | American Journal of Agricultural Economics | Q1* in Economics | Other thematic areas: Q1* in Agricultural and Biological Sciences |
| 1991 | None |  |  |
| 1992 | Annals of the American Academy of Political and Social Science,  Quarterly Journal of Economics | Q1* in Social Sciences  Q1* in Economics | Specific category: Q1* in Social Sciences  Specific category: Q1* in Economics and Econometrics |
| 1993 | None |  |  |
| 1994 | None |  |  |
| 1995 | Economica | Q1* in Economics | Specific category: Q1* in Economics and Econometrics |
| 1996 | Oxford Economic Papers | Q2* in Economics | Specific category: Q2* in Economics and Econometrics |
| 1997 | None |  |  |
| 1998 | Social Forces | Q1* in Social Sciences | Specific categories: Q1* in Sociology and Political Science; Development |
| 1999 | Jahrbucher fur Nationalokonomie und Statistik,  Monthly Labor Review | Q2* in Economics  Q4* in Business | Specific category: Q1* in Economics and Econometrics  Specific categories: Q4* in Organizational Behavior and Human Resource Management, Strategy and Management |
| 2000 | Professional Geographer | Q1* in Social Sciences | Other thematic areas: Q2* in Earth and Planetary Sciences |
| 2001 | None |  |  |
| 2002 | South African Journal of Economics | Q2 in Economics | Specific category: Q2 in Economics and Econometrics |
| 2003 | None |  |  |
| 2004 | Economic Record,  Review of Economic Studies | Q2 in Economics  Q1 in Economics | Specific category: Q2 in Economics and Econometrics  Specific category: Q1 in Economics and Econometrics |
| 2005 | Kyklos | Q1 in Economics | Specific category: Q1 in Economics and Econometrics |
| 2006 | Journal of Economics/ Zeitschrift fur Nationalokonomie | Q3 in Economics | Specific category: Q3 in Economics and Econometrics |
| 2007 | Manchester School,  Metroeconomica,  Proceedings of the National Academy of Sciences of the United States of America | Q2 in Economics  Q2 in Economics  Not classified in any core | Specific category: Q2 in Economics and Econometrics  Specific category: Q2 in Economics and Econometrics  Q1 in Multidisciplinary |
| 2008 | None |  |  |
| 2009 | Annals of Occupational Hygiene,  Occupational Medicine,  Public Health Reports | Not classified in any core  Not classified in any core  Not classified in any core | Q2 in Environmental Science (Publich Health; Environmental and Occupational Health)  Q2 in Medicine (Publich Health; Environmental and Occupational Health)  Q2 in Medicine (Publich Health; Environmental and Occupational Health) |
| 2010 | Current Science,  Journal of Hydrology,  Plant and Soil,  Review of Income and Wealth | Not classified in any core  Not classified in any core  Not classified in any core  Q2 in Economics | Q2 in Multidisciplinary  Q1 in Earth and Planetary Sciences; Environmental Science  Q1 in Agricultural and Biological Sciences  Specific category: Q2 in Economics and Econometrics |
| 2011 | American Journal of Economics and Sociology,  Chemical and Engineering News,  Economic History Review,  International Journal of Comparative Sociology | Q2 in Economics  Not classified in any core  Q1 in Economics  Q1 in Social Sciences | Other thematic areas: Q2 in Social Sciences  Professional and technical weekly news magazine  Other thematic areas: Q1 in Arts and Humanities  Specific categories: Q2 Sociology and Political Sciences, Social Sciences |
| 2012 | American Behavioral Scientist,  Bulletin of Economic Research | Q1 in Social Sciences  Q2 in Economics | Specific category: Q1 in Sociology and Political Sciences  Specific category: Q2 in Economics and Econometrics |
| 2013 | Agrekon,  Global Change Biology,  Labor History,  Philosophical transactions. Series A, Mathematical, physical, and engineering sciences | Q2 in Economics  Not classified in any core  Q1 in Social Sciences  Not classified in any core | Other thematic areas: Q2 in Agricultural and Biological Sciences  Q1 in Environmental Science; Agricultural and Biological Sciences; Earth and Planetary Sciences  Other thematica areas: Q1 in History  Q1 in Engineering; Physics and Astronomy |
| 2014 | Journal of Agricultural Economics,  Norsk Geografisk Tidsskrift | Q2 in Economics  Q2 in Social Sciences | Other thematic areas: Q1 in Agricultural and Biological Sciences  Other thematica areas: Q2 in Earth and Planetary Sciences |
| 2015 | Annals of Public and Cooperative Economics,  De Economist | Q2 in Economics  Q2 in Economics | Other thematic areas: Q2 in Social Sciences  Specific category: Q2 in Economic and Econometrics |
| 2016 | British Food Journal,  International Journal of Production Research,  Journal of Agricultural Science,  Kexue Tongbao/Chinese Science Bulletin | Q2 in Business  Q1 in Business  Not classified in any core  Not classified in any core | Other thematic areas: Q2 in Agricultural and Biological Sciences  Other thematic areas: Q1 in Engineering  Q2 in Agricultural and Biological Sciences  Q2 in Multidisciplinary |
| 2017 | International Review of Education,  Journal of Applied Psychology,  Teoriya i Praktika Fizicheskoy Kultury | Not classified in any core  Q1 in Business; Industrial Relations; Organizational Behavior and HRM  Q4 in Social Sciences | Q1 in Education  Other tematic areas: Q1 in Psychology  Other thematic areas: Q4 in Education |
| 2018 | Atmospheric Environment,  Business Horizons,  Human Relations,  Human Resource Management,  Journal of Economic History,  Revista brasileira de enfermagem | Not classified in any core  Q1 in Business  Q1 in Business; Social Sciences; Organizational Behavior and HRM  Q1 in Business; Organizational Behavior and HRM  Q1 in Economics  Not classified in any core | Q1 in Environmental Science; Earth and Planetary Sciences; Chemical Engineering  Specific categories: Q1 in Business and International Management; Management of Technology and Innovation  Specific category: Q1 in Organizational Behavior and HRM  Specific category: Q1 in Organizational Behavior and HRM  Other thematic areas: Q1 in History  Q3 in Nursing |
| 2019 | British Journal of Sociology,  Journal of Asian and African Studies,  Journal of Sociology,  Socio-Economic Planning Sciences | Q1 in Social Sciences  Q2 in Social Sciences; Development  Q2 in Social Sciences  Q1 in Business; Economics; Social Sciences; Development | Specific category: Q1 in Sociology and Political Sciences  Specific categories: Q2 in Development; Geography, Planning and Development  Specific category: Q2 in Sociology and Political Sciences  Specific categories: Q1 in Management Science and Operations Research; Development |
| 2020 | Meditsina Truda I Promyshlennaya Ekologiya,  Voprosy Istorii,  Water Research | Not classified in any core  Not classified in any core  Not classified in any core | Q4 in Medicine; Environmental Science (Public Health, Environmental and Occupational Health; Industrial and Manufacturing Engineering)  Q4 in History  Q4 in Environmental Science; Chemical Engineering |
| 2021 | Australian Economic Papers,  Business History,  Foreign Trade Review,  Fuel,  IEEE Transactions on Engineering Management,  Journal of Experimental Botany,  Journal of Financial and Quantitative Analysis,  Nature,  Physiologia Plantarum,  Solar Energy | Q2 in Economics  Not classified in Economics  Q2 in Economics  Not classified in any core  Q1 in Business  Not classified in any core  Q1 in Economics  Not classified in any core  Not classified in any core  Not classified in any core | Specific category: Q2 in Economics and Econometrics  Other thematic areas: Q1 in History  Specific category: Q2 in Economics and Econometrics  Q1 in Energy; Chemical Engineering  Other thematic areas: Q1 in Engineering (Management Science and Operations Research; Strategy and Management)  Q1 in Plant Science  Specific category: Q1 in Economics and Econometrics  Q1 in Multidisciplinary  Q1 in Plant Science  Q1 in Energy; Engineering |
| 2022 | Acta Medica Philippina,  Comparative Political Studies,  Science Progress,  Ugol,  Water, Air, and Soil Pollution | Not classified in any core  Q1 in Social Sciences  Not classified in any core  Not classified in any core  Not classified in any core | Q3 in Medicine  Specific category: Q1 in Sociology and Political Sciences  Q3 in Multidisciplinary  Q4 in Energy; Engineering  Q2 in Environmental Science |
| 2023 | Geological Journal,  ICES Journal of Marine Science,  International Journal of Environmental Studies,  Journal of Agricultural and Food Chemistry,  Journal of the Science of Food and Agriculture,  New Phytologist,  Plant Physiology,  Sociologia Ruralis | Not classified in any core  Not classified in any core  Q2 in Social Sciences  Not classified in any core  Not classified in any core  Not classified in any core  Not classified in any core  Q1 in Social Sciences; Development | Q2 in Earth and Planetary Sciences; Environmental Science  Q1 in Environmental Science  Other thematic areas: Q2 in Earth and Planetary Sciences; Environmental Science (Geography, Planning and Development)  Q1 in Agricultural and Biological Sciences; Chemistry; Environmental Science  Q2 in Agricultural and Biological Sciences (Food Science; Agronomy and Crop Science)  Q1 in Agricultural and Biological Sciences  Q1 in Agricultural and Biological Sciences  Specific categories: Q1 in Sociology and Political Science; Development; Demography |
| 2024 | Acta Psychologica,  Chemical Engineering Science,  Higher Education Quarterly,  Human Ecology,  Inquiry (United States),  International Journal of Biometeorology,  International Journal of Management Education,  Journal of Agronomy and Crop Science,  Journal of Chemical Education,  Journal of Economic Entomology,  Journal of Finance,  Ocean Engineering,  Physics of Fluids,  Plant Growth Regulation,  Public Health,  Research in Higher Education,  Scandinavian Journal of Public Health | Not classified in any core  Not classified in any core  Q1 in Social Sciences  Q1 in Social Sciences  Not classified in any core  Not classified in any core  Q1 in Business; Social Sciences  Not classified in any core  Not classified in any core  Not classified in any core  Q1 in Economics  Not classified in any core  Not classified in any core  Not classified in any core  Not classified in any core  Not classified in any core  Not classified in any core | Q2 in Psychology; Q3 in Education  Q1 in Chemical Engineering; Chemistry; Engineering (Industrial and Manufacturing Engineering)  Other thematic areas: Q1 in Education  Other thematic areas: Q1 in Environmental Science  Q2 in Medicine; Health Profession  Q1 in Environmental Science, Medicine, Agricultural and Biological Sciences  Specific category: Q1 in Strategy and Management  Q1 in Agricultural and Biological Sciences  Q4 in Chemistry; Education  Q1 in Agricultural and Biological Sciences; Q2 in Environmental Science  Specific categories: Q1 in Accounting; Economics and Econometrics; Finance  Q1 in Engineering; Environmental Science  Q2 in Physics and Astronomy  Q1 in Agricultural and Biological Sciences  Q2 in Medicine (Public Health, Environmental and Occupational Health)  Q1 in Education  Q1 in Medicine (Public Health, Environmental and Occupational Health) |
| *: This symbol indicates the current quartile of the journal. We used current quartiles as a proxy for the years before 1999, when the Scimago Journal Rank (SJR) didn't exist.  Core thematic areas relevant to the SDG 08 in the Scimago classification:  Economics, Econometrics, and Finance (Thematic area or Major Subject Category): marked as Economics  Business, Management and Accounting (Thematic area or Major Subject Category): marked as Business  Social Sciences (Thematic area or Major Subject Category)  Development (Specific category or Minor Subject Category)  Organizational Behavior and Human Resource Management (Specific category or Minor Subject Category): marked as Organizational Behavior and HRM  Industrial Relations (Specific category or Minor Subject Category) | | | |

**Table 3.** SDG 13 (*Climate Action*)

| **Year** | **Established list** | **Quartile Rank for core relevant areas in SJR** | **Quartiles in other thematic areas (or specific minor categories)** |
| --- | --- | --- | --- |
| 1990 | Nature,  Transactions of the American Fisheries Society | Not classified in E.S.  Not classified in E.S. | Q1* in Multidisciplinary |
| 1991 | None |  |  |
| 1992 | None |  |  |
| 1993 | None |  |  |
| 1994 | Eos,  Fuel | Not classified in E.S.  Not classified in E.S. | Scientific newsmagazine - Transaction Journal  Q1* in Applied Chemistry and Chemical Engineering |
| 1995 | None |  |  |
| 1996 | None |  |  |
| 1997 | Proceedings of the National Academy of Sciences of the United States of America,  Science | Not classified in E.S.  Not classified in E.S. | Q1* in Multidisciplinary  Q1* in Multidisciplinary |
| 1998 | None |  |  |
| 1999 | None |  |  |
| 2000 | None |  |  |
| 2001 | None |  |  |
| 2002 | ACS Symposium Series,  Journal of Hydrology | Not classified in E.S.  Q1* in Environmental Science | Conference Proceedings - Book series  Specific category: Q1* in Water Science and Technology |
| 2003 | None |  |  |
| 2004 | Ibis | Not classified in E.S. | Q1 in Agricultural and Biological Sciences |
| 2005 | Canadian Journal of Agricultural Economics,  Journal of the American Chemical Society | Not classified in E.S.  Not classified in E.S. | Q1 in Chemistry  Q1 in Agricultural and Biological Sciences |
| 2006 | Chemie-Ingenieur-Technik,  Journal of the Meteorological Society of Japan,  Philosophical transactions. Series A: Mathematical, physical, and engineering sciences | Not classified in E.S.  Q2 in Meteorology and Atmospheric Sciences  Not classified in E.S. | Q1 in Chemistry  Specific category under Physics and Astronomy  Q1 in Engineering, Mathematics, Physics and Astronomy |
| 2007 | AIChE Journal,  Chemical Engineering Science,  Soil Science and Plant Nutrition | Not classified in E.S.  Not classified in E.S.  Not classified in E.S. | Q1 in Chemical Engineering  Q1 in Chemical Engineering  Q1 in Agricultural and Biological Sciences |
| 2008 | Current Science,  New Phytologist,  Political Science,  Proceedings of the Royal Society B: Biological Sciences | Not classified in E.S.  Not classified in E.S.  Not classified in E.S.  Q1 in Environmental Science | Q2 in Multidisciplinary  Q1 in Agricultural and Biological Sciences  Q2 in Social Sciences  Specific categories: Q1 in Ecology, Evolutionary Biology, Conservation Science |
| 2009 | Hydrobiologia,  Solar Energy | Q1 in Environmental Science  Q1 in Environmental Science | Specific category: Q1 in Acquatic Science  Specific categories: Q1 in Energy Engineering and Power Technology, Renewable Energy, Sustainability and the Environment |
| 2010 | Annals of the Association of American Geographers,  Chemical and Engineering News,  Geochimica et Cosmochimica Acta,  Journal of Agricultural Science,  Journal of Experimental Biology,  Leading Edge,  Philosophical Transactions of the Royal Society B: Biological Sciences | Q1 in Environmental Science  Not classified in E.S.  Q1 in Environmental Science  Not classified in E.S.  Q1 in Environmental Science  Not classified in E.S.  Q1 in Environmental Science | Specific category: Q1 in Geography, Planning and Development  Scientific news magazine  Specific category: Q1 in Geochemistry and Petrology  Q1 in Agricultural and Biological Sciences  Other thematic areas: Q1 in Agricultural and Biological Sciences; Biochemistry, Genetics and Molecular Biology; Neuroscience  Q3 in Earth and Planetary Science (Geology and Geophysics)  Other thematic areas: Q1 in Agricultural and Biological Sciences; Biochemistry, Genetics and Molecular Biology; Immunology and Microbiology |
| 2011 | Combustion and Flame,  Euphytica,  ICES Journal of Marine Science,  Journal of Forestry,  Marine and Freshwater Research | Not classified in E.S.  Not classified in E.S.  Q1 in Environmental Science  Q1 in Environmental Science  Q2 in Environmental Science | Q1 in Engineering  Q1 in Agricultural and Biological Science  Specific categories: Q1 in Aquatic Science; Ecology, Evolution, Behavior and Systematics; Oceanography; Fisheries  Specific categories: Q1 in Forestry; Geology; Soil Science  Specific category: Q2 in Aquatic Science |
| 2012 | Annals of Botany,  Bulletin of the Atomic Scientists,  Journal of Dairy Science | Not classified in E.S.  Not classified in E.S.  Not classified in E.S. | Q1 in Agricultural and Biological Sciences  Scientific news magazine  Q1 in Agricultural and Biological Sciences |
| 2013 | American Journal of Botany,  Electrochimica Acta,  Inorganic Chemistry,  Journal of Animal Science,  Journal of Experimental Botany | Not classified in E.S.  Not classified in E.S.  Not classified in E.S.  Not classified in E.S.  Q1 in Environmental Science | Q1 in Agricultural and Biological Sciences  Q1 in Chemical Engineering  Q1 in Chemistry  Q1 in Agricultural and Biological Sciences  Other thematic areas: Q1 in Agricultural and Biological Sciences; Biochemistry, Genetics and Molecular Biology |
| 2014 | Carbon,  Journal of Chemical and Engineering Data | Not classified in E.S.  Not classified in E.S. | Q1 in Materials Science  Q2 in Chemical Engineering |
| 2015 | Canadian Journal of Chemical Engineering,  Chimia,  Geophysics,  Houille Blanche,  Journal of the Electrochemical Society,  Kexue Tongbao/Chinese Science Bulletin | Not classified in E.S.  Not classified in E.S.  Not classified in E.S.  Q3 in Environmental Science  Q2 in Environmental Science  Not classified in E.S. | Q2 in Chemical Engineering  Q2 in Chemistry  Q3 in Earth and Planetary Sciences  Specific category: Q3 in Water Science and Technology  Other thematic areas: Q2 in Energy  Q2 in Multidisciplinary |
| 2016 | None |  |  |
| 2017 | International Journal of Heat and Mass Transfer,  Journal of Colloid and Interface Science,  Journal of the Science of Food and Agriculture,  Polymer | Not classified in E.S.  Not classified in E.S.  Not classified in E.S.  Not classified in E.S. | Q1 in Energy (Thermodynamics; Energy Engineering and Power Technology; Materials Science)  Q1 in Chemistry (Materials Science)  Q1 in Agricultural and Biological Science  Q1 in Materials Science |
| 2018 | Journal of Materials Science,  Journal Physics D: Applied Physics | Not classified in E.S.  Not classified in E.S. | Q1 in Materials Science  Q1 in Physics and Astronomy (Applied Physiscs, Electric and Electric Engineering) |
| 2019 | Annals of the New York Academy of Sciences,  Journal of Quantitative Spectroscopy and Radiative Transfer,  Marine Geology,  Monthly Notices of the Royal Astronomical Society,  Physiologia Plantarum | Not classified in E.S.  Q1 in Environmental Science  Q2 in Environmental Science  Not classified in E.S.  Not classified in E.S. | Q1 in Multidisciplinary  Other thematic areas: Q1 in Earth and Planetary Sciences  Other thematic areas: Q2 in Earth and Planetary Sciences  Q1 in Physics and Astronomy  Q1 in Agricultural and Biological Sciences |
| 2020 | Analytical Chemistry,  Chemical Physics Letters,  International Journal of Production Research,  Journal of Applied Polymer Science,  Journal of Solid-State Chemistry,  Materials Research Bulletin,  Urban Studies | Not classified in E.S.  Not classified in E.S.  Not classified in E.S.  Not classified in E.S.  Not classified in E.S.  Not classified in E.S.  Q1 in Environmental Science | Q1 in Chemistry  Q2 in Chemistry  Q1 in Engineering (Industrial and Manufacturing Engineering, Management Science and Operations Research)  Q2 in Materials Science  Q2 in Materilas Science  Q1 in Materials Science  Specific category: Q1 in Environmental Studies |
| 2021 | Biological Reviews,  Combustion Science and Technology,  European Journal of Inorganic Chemistry,  Geothermics,  International Journal of Rock Mechanics and Mining Sciences,  Journal of Chemical Physics,  Marine Technology Society Journal,  Pure and Applied Geophysics,  Socio-Economic Planning Sciences,  World Oil | Not classified in E.S.  Not classified in E.S.  Not classified in E.S.  Q2 in Environmental Science  Q1 in Environmental Science  Not classified in E.S.  Not classified in E.S.  Not classified in E.S.  Not classified in E.S.  Not classified in E.S. | Q1 in Agricultural and Biological Sciences  Q2 in Engineering (Aerospace Engineering, Combustion, Energy Engineering in Power Technology)  Q2 in Chemistry  Specific categories: Q1/Q2 Renewable Energy, Sustainability and the Environment; Economic Geology; Geotechnical Engineering and Engineering Geology  Other thematic areas: Q1 in Engineering; Earth and Planetary Sciences  Q1 in Chemistry  Q3 in Engineering (Ocean Engineering)  Q3 in Earth and Planetary Sciences  Q1 in Social Sciences  Q2 in Energy (Fuel Technology and Petroleum) |
| 2022 | AAPG Bulletin,  Anais da Academia Brasileira de Ciencias,  Bautechnik,  Canadian Mining Journal,  Cement and Concrete Research,  Economic Computation and Economic Cybernetics Studies and Research,  JOM,  Journal of Molecular Structure,  Journal of Physics and Chemistry of Solids,  Professional Geographer,  SPE Journal | Q1 in Environmental Science  Not classified in E.S.  Not classified in E.S.  Not classified in E.S.  Not classified in E.S.  Not classified in E.S.  Not classified in E.S.  Not classified in E.S.  Not classified in E.S.  Q2 in Environmental Science  Not classified in E.S. | Other thematic areas: Q1 in Earth and Planetary Sciences  Q2 in Multidisciplinary  Q2 in Engineering (Building and Construction)  Scientific news magazine  Q1 in Materials Science  Q2 in Computer Science  Q2 in Materials Science  Q2 in Chemistry  Q2 in Physics and Astronomy  Other thematic areas: Q2 in Social Sciences  Q2 in Energy (Fuel Technology and Petroleum) |
| 2023 | Archives of Agronomy and Soil Science,  Beton- und Stahlbetonbau,  Engineering Structures,  Forestry,  Geological Journal,  Geophysical Journal International,  Magazine of Concrete Research,  Management Science,  Nederlands Tijdschrift voor Geneeskunde,  Plant Growth Regulation,  Plant Physiology,  Thermal Engineering | Not classified in E.S.  Not classified in E.S.  Not classified in E.S.  Q1 in Environmental Science  Q2 in Environmental Science  Not classified in E.S.  Not classified in E.S.  Not classified in E.S.  Not classified in E.S.  Not classified in E.S.  Not classified in E.S.  Not classified in E.S. | Q1 in Agricultural and Biological Sciences  Q3 in Engineering (Building and Construction, Civil and Structural Engineering)  Q1 in Engineering (Civil and Structural Engineering)  Specific category: Q1 in Forestry  Other thematic areas: Q2 in Earth and Planetary Sciences  Q1 in Earth and Planetary Sciences  Q2 in Engineering (Building and Construction, Civil and Structural Engineering, Materials Science)  Q1 in Business, Management and Accounting  Q3 in Medicine  Q1 in Agricultural and Biological Sciences  Q1 in Agricultural and Biological Sciences  Q2 in Energy (Energy Engineering and Power Technology) |
| 2024 | American Journal of Agricultural Economics,  Annals of Regional Science,  Appetite,  Chemical Papers,  Communications in Soil Science and Plant Analysis,  Computers and Electrical Engineering,  IEEE Transactions on Engineering Management,  Indian Geotechnical Journal,  Journal of Agricultural and Food Chemistry,  Journal of Asian and African Studies,  Journal of Chemical Education,  Journal of Economic Entomology,  Journal of Pest Science,  Journal of Phycology,  Journal of the Royal Society of New Zealand,  Kybernetes,  Nuclear Engineering and Design,  Physica Scripta,  Physics of Fluids,  Potato Research,  Quality and Quantity,  Quarterly Journal of the Royal Meteorological Society,  Theoretical And Applied Genetics,  Transportation | Not classified in E.S.  Q2 in Environmental Science  Not classified in E.S.  Q2 in Environmental Science  Not classified in E.S.  Not classified in E.S.  Not classified in E.S.  Not classified in E.S.  Q1 in Environmental Science  Not classified in E.S.  Not classified in E.S.  Q2 in Environmental Science  Q1 in Environmental Science  Q1 in Environmental Science  Q2 in Environmental Science  Not classified in E.S.  Q1 in Environmental Science  Not classified in E.S.  Not classified in E.S.  Not classified in E.S.  Not classified in E.S.  Q1 Meteorology and Atmospheric Sciences  Not classified in E.S.  Q1 in Environmental Science | Q1 in Angricultural and Biological Sciences  Other thematic areas: Q2 in Social Sciences  Q1 in Psychology (Psychology, Nutrition adn Dietetics)  Other thematic areas: Q2 in Chemistry  Q2 in Agricultural and Biological Sciences  Q1 in Computer Science  Q1 in Business, Management, And Accounting; Engineering  Q3 in Earth and Planetary Sciences  Other thematic areas: Q1 in Agricultural and Biological Sciences; Chemistry  Q2 in Social Sciences  Q2 in Chemistry  Other thematic areas: Q2 in Agricultural and Biological Sciences  Other thematic areas: Q1 in Agricultural and Biological Sciences  Other thematic areas: Q1 in Agricultural and Biological Sciences  Specific category: Q2 in Environmenal Studies  Q1 in Computer Science, Engineering, Social Sciences  Other thematic areas: Q1 in Engineering; Energy; Materials Science; Physics and Astronomy  Q3 in Physics and Astronomy  Q1 in Physics and Astronomy  Q3 in Agricultural and Biological Sciences  Q2 in Social Sciences  Specific category under Physics and Astronomy  Q1 in Agricultural and Biological Sciences  Other thematic areas: Q1 in Social Sciences |
| *: This symbol indicates the current quartile of the journal. We used current quartiles as a proxy for the years before 1999, when the Scimago Journal Rank (SJR) didn't exist.  Core thematic areas relavant to the SDG 13 in the Scimago classification:  Environmental Science (Thematic area or Major Subject Category);  Meteorology and Atmospheric Science (Minor Subject Category) | | | |
